# Impact of cannabinoids on cancer outcomes in patients receiving immune checkpoint inhibitor immunotherapy

**DOI:** 10.3389/fimmu.2025.1497829

**Published:** 2025-03-05

**Authors:** MariaLuisa Vigano, Lixing Wang, Alia As’sadiq, Suzanne Samarani, Ali Ahmad, Cecilia T. Costiniuk

**Affiliations:** ^1^ Division of Experimental Medicine, Faculty of Medicine and Health Sciences, McGill University, Montreal, QC, Canada; ^2^ Infectious Diseases and Immunity in Global Health Program, Research Institute of the McGill University Health Centre, Montreal, QC, Canada; ^3^ Department of Microbiology and Immunology, McGill University, Montreal, QC, Canada; ^4^ Division of Infectious Diseases and Chronic Viral Illnesses Service, McGill University Health Centre, Montreal, QC, Canada

**Keywords:** cancer, cannabinoids, cannabinoid receptors, immune checkpoints, immune checkpoint inhibitor immunotherapy

## Abstract

Cannabinoids relieve pain, nausea, anorexia and anxiety, and improve quality of life in several cancer patients. The immunotherapy with checkpoint inhibitors (ICIs), although very successful in a subset of patients, is accompanied by moderate to severe immune-related adverse events (ir-AE) that often necessitate its discontinuation. Because of their role in symptomatic relief, cannabinoids have been used in combination with immune checkpoint inhibitor (ICI) immunotherapy. A few studies strongly suggest that the use of medicinal cannabis in cancer patients attenuates many of the ir-AE associated with the use of ICI immunotherapy and increase its tolerability. However, no significant beneficial effects on overall survival, progression free survival or cancer relapses were observed; rather, some of the studies noted adverse effects of concurrent administration of cannabinoids with ICI immunotherapy on the clinical benefits of the latter. Because of cannabinoids’ well documented immunosuppressive effects mediated through the cannabinoid recptor-2 (CB2), we propose considering this receptor as an inhibitory immune checkpoint *per se*. A simultaneous neutralization of CB2, concurrent with cannabinoid treatment, may lead to better clinical outcomes in cancer patients receiving ICI immunotherapy. In this regard, cannabinoids such as cannabidiol (CBD) and cannabigerol (CBG), with little agonism for CB2, may be better therapeutic choices. Additional strategies e.g., the use of monoacylglycerol lipase (MAGL) inhibitors that degrade some endocannabinoids as well as lipogenesis and formation of lipid bilayers in cancer cells may also be explored. Future studies should take into consideration gut microbiota, CYP450 polymorphism and haplotypes, cannabinoid-drug interactions as well as genetic and somatic variations occurring in the cannabinoid receptors and their signaling pathways in cancer cells for personalized cannabis-based therapies in cancer patients receiving ICIs. This may lead to rational knowledge-based regimens tailored to individual cancer patients.

## 1 Introduction

Cancer, resulting from abnormal and uncontrolled proliferation of cells in the body, is the leading cause of death in humans. Globally, it caused nearly 20 million deaths in 2020 (https://www.who.int/news-room/fact-sheets/detail/cancer; accessed on 7 June 2024). There exist more than 100 types of cancer affecting different tissues and cell types, and resulting from different etiology and carcinogenetic processes. A wide variety of chemotherapeutic drugs have been developed with variable efficacies in different types of cancers. Due to cancers’ remarkable ability to develop resistance to anti-cancer chemotherapy as well as to evade body’s anti-cancer immune responses, search for novel, more effective and safer anti-cancer drugs is ever ongoing. In this regard, immune checkpoint inhibitors (ICIs), also known as immune checkpoint blockers (ICBs), represent a novel class of anti-cancer therapeutics that aim at invigorating anti-cancer immune responses in cancer patients. Immune cells express different checkpoints to inhibit body harm that may be caused by an overactive immune response (2, 3). However, the inhibition of the checkpoints in cancer patients is accompanied by many moderate to severe immune-related adverse events (ir-AE). To overcome these adverse events and improve the tolerability of ICIs, clinicians/researchers are resorting to the use of cannabinoids. It is noteworthy that cancer patients are also often prescribed cannabinoids for relief from cancer/chemotherapy-associated pain, anxiety, nausea, anorexia and insomnia. Furthermore, current legalization of medicinal cannabis in many countries in the World as well in many states in the USA has provided impetus to its use for both medicinal and recreational purposes. This review is focused to understand pros and cons of the use of cannabinoids in cancer patients undergoing ICI immunotherapy. To understand the complex interplay between ICIs and cannabinoids, we will first describe different immune checkpoints, their ligands, relevant ICIs and their potential mechanisms of action for activating anti-cancer immunity. Thereafter, we will review cannabinoids and their known receptors through which they exert their effects on immune and nonimmune cells as well as on cancer cells in the human body. We will also present pre-clinical and clinical evidence regarding the interaction of ICIs and cannabinoids in the tumor bearing animal models and cancer patients. Finally, we will discuss various factors that are known to regulate effects of cannabinoids on immunotherapy with ICIs in these patients.

## 2 Immunotherapy with immune checkpoint inhibitors in cancer patients

ICIs have been hailed as game changer in the treatment of cancer; they have drastically changed the landscape of anti-cancer therapy (2, 3). ICIs-based immunotherapies have revolutionized therapies for solid tumors including melanoma, non-small cell lung cancer (NSCLC), renal cell carcinoma (RCC) and breast cancer, to mention a few (2, 3). In the context of cancer immunotherapy, immune checkpoints refer to key molecules that negatively regulate activation and effector functions of immune cells. They include receptors or co-receptors that are expressed on immune cells (T, B, Natural Killer and dendritic cells, and macrophages) and, upon engagement with their respective ligands or counter-receptors, negatively regulate the immune cell functions such as cytotoxicity, phagocytosis, production of cytokines and chemokines, etc. Additionally, they also promote the development and function of immunosuppressive CD4^+^ T regulatory cells (Treg) cells and myeloid-derived suppressor cells (MDSC; pathologically activated monocytes and neutrophils) (4). Under physiological conditions, they are not expressed in immune cells, however, their expression occurs upon activation and persistent antigenic stimulation. They serve as homeostatic mechanisms to protect the host from harmful tissue destructive effects of an activated and over-functional immune system. They negatively regulate effector functions of immune cells, and the immune cells expressing these checkpoints are referred to as “exhausted”. Cancer cells exploit these homeostatic mechanisms, and express ligands for the immune checkpoints in order to suppress and evade anti-cancer immune responses. Furthermore, immune and other cells present in the tumor microenvironment (TME) such as Bregs, Tregs, MDSC, cancer-associated macrophages and fibroblasts express ligands/counter receptors for the checkpoints.

The checkpoints include Cytotoxic T Lymphocyte Antigen (CTLA)-4, Programmed Death (PD)-1, Lymphocyte-Activation Gene (LAG)-3, T-Cell Immunoglobulin and Mucin-domain containing (TIM-3), V-domain Immunoglobulin Suppressor of T cell Activation (VISTA) and others (listed in **Table 1**) (3, 5). The immune checkpoints are currently being targeted through monoclonal antibodies (mAb) each of which targets a specific checkpoint. The mAb are humanized i.e., all or most of their murine sequences, except for their checkpoint binding sites, are replaced by the corresponding human sequences. The humanization reduces their antigenicity when administered to cancer patients. The mAb of IgG4 isotype lack FcR-mediated effector functions and work by inhibiting interaction of the targeted checkpoint with its ligand/counter-receptor through steric hinderance. Thus, through inhibiting interaction between a checkpoint and its ligand and/or by eliminating checkpoint or its counter-receptor bearing immunosuppressive immune cells and cancer cells, these humanized antibodies release the immune cells from inhibitory effects of the checkpoint, and augment their anti-tumor effector functions. The mAb of IG1 isotype, in addition to inhibiting a checkpoint-ligand interaction, can also mediate killing of the checkpoint ligand-positive cancer cells through antibody-dependent cell-mediated cytotoxicity (ADCC) by CD16+ NK cells and non-classical CD16+ monocytes. Alternately, these antibodies can also induce killing of the cancer cells through complement activation. They could also induce phagocytosis of the cells through antibody-dependent cellular phagocytosis (ADCP) by macrophages. The humanized antibodies are commonly referred to as immune checkpoint inhibitors (ICIs) or immune checkpoint blockers (ICBs). A list of currently targeted inhibitory immune checkpoints, their ligands/counter-receptors as well as their specific ICIs (approved and/or under clinical trials) is provided in **Table 1**.

**Table 1:** Immune checkpoints, their counter-receptors and inhibitors.

Checkpoint	Ligand/Counter Receptor	Expression	Mechanism of ICIs	Inhibitors (Isotype), the year when approved	References
CTLA-4 (CD152)	Binds with CD80 (B7.1) and CD86 (B7.2) with higher affinity and avidity as compared with CD28	Activated T cells, Treg, Bregs, NK cells	Inhibits CTLA-4 interaction with CD80 and CD86, permitting CD28-mediated co-stimulatory signals to T cells; Enhances T cell activation; Induces apoptosis of Tregs; Prevents trogocytosis of CD80 and CD86 on surrounding APC	Ipilumab (IgG1), 2011; Tremelimumab (IgG2), 2022	(3, 170, 171)
PD-1 (CD279)	PDL-1 (CD274), PDL-2 (CD273)	Mainly by activated T cells, also by NK, B and myeloid cells	Inhibits PD-1/PDL-1 interaction between T cells and cancer cells/myeloid cells in TME; Increases T cell activation; inhibits effector T cell anergy	Nivolumab IgG4), 2014; Pembrolizumab (IgG4), 2014; Cemiplimab (IgG4), 2018; Dostarlimab (IgG4), 2023	(2, 171)
PDL-1 or B7-H1 (CD274)	PD-1	PDL-1 expressed on cancer cells as well as on myeloid ells in the TME	Induces phagocytosis and ADCC of cancer cells; through inhibiting PD-1/PDL-1 interaction, promotes effector T cell activation and reduces effector T cell anergy and Treg differentiation	Avelumab (IgG1), 2017; Atezolizumab (IgG1), 2016; Durvalumab (IgG1), 2016	(172)
TIM-3 or HAVCR2 (CD366)	PS, CEACAM (CD66a), Gal-9, HMGB-1	Th1, Th17, Tregs, T8, NK, DC, monocytes and macrophages; Cancer cells express ligands: Gal-9, CEACAM	Inhibits TIM-3 on T cells with Gal-9 on cancer cells and other cells in TME; Inhibits TIM3 interaction on T cells with HMGB1 on other immune cells; Prevents T cell exhaustion; Promotes recognition of nucleic acids in endosomes through preventing TIM-3 binding with HMGB-1; TIM-3 prevents phagocytosis of apoptotic cells by macrophages and CD8+ DC	Cobolimab (IgG4), Sabatomimab (IgG4)	(173)
TIGIT	PVR (CD155), PVRL2 (CD112, Nectin-2), PVRL3	T4, T8, Treg, NK; Cancer cells and APC in TME express PVR, PVR-L2	Inhibits TIGIT on immune cells with TIGIT ligands on cancer cells and non-cancer cells in TME; Increases T cell activation and Treg inactivation	Vibostolimab (IgG1); Ociperlimab (IgG1); Tiragolumab (IgG1), 2021; Domvanalimab (IgG1)	(174)
LAG-3 (CD223)	MHC-II, FGL-1, Gal-3, LSECTIN	T4, T8, B, NK, pDC; Cancer cells express ligands: FGL-1 and Gal-3	Inhibits LAG-3 on T cells, B cells, pDC and NK cells with LAG-3 ligands expressed on cancer cells; Increases effector functions of T, NK and B cells; Increases innate immunity through activation of pDC	Fianlimab (IgG4); Ieramilimab (IgG4); Relatlimab (IgG4), 2022	(175)
VISTA (B7-H5; a PD-1 homolog)	VISTA, VISG3, VISG8, PSLG1, LIRG1	VISTA is expressed on naïve T cells, NK cells, Tregs, monocytes, macrophages, DC and neutrophils; VISTA and its ligands are also overexpressed on cancer cells (in particular on cancer stem cells), tumor-infiltrated myeloid cells and Tregs in TME; VISTA can interact with itself and with its ligands in trans as well as in cis	VISTA blocker inhibits interaction of VISTA on cancer with VISTA on immune cells, as well as with other VISTA receptors; Promotes anti-cancer effector functions of immune cells; inhibits cancer stem cell survival and cancer progression; Reduces Tregs through ADCC; Increases progenitor and memory-like CD8+ T cells; Enhances anti-cancer vaccine responses	Onvatilimab (IgG1) CI-8993 (IgG1)	(176–178)
NKG2A (expressed as a heterodimer with CD94)	HLA-E	Subsets of NK and CD8+ T cells; Cancer cells over-express HLA-E	Inhibits interaction between HLA-E on cancer with NKG2A on NK and CD8+ T cells and increases anti-cancer activity of these cytolytic immune cells	Monalizumab (IgG4), 2009	(179, 180)
ILT-2 (LILRB-1 or CD85J), ILT-4 (LILRB-2 or CD85d)	MHC-class I with higher affinity for HLA-G	IL-T2 is expressed on NK, CD8+ T and B cells, monocytes, macrophages, MDSC and DC; ILT-4 expression is restricted to myeloid cells; Cancer cells over-express HLA-G	ICI inhibit ILT-2 and ILT-4 mediated inhibition of NK and CD8+ T cells as well as of myeloid cells; Reprogram myeloid cells in the TME; Promote phagocytosis of cancer cells and antigen presentation by DC; Increases cancer cell radio-sensitivity	BND-22 (IgG4) for ILT2; MK-4830 (IgG4) for ILT-4; Simultaneous blockade of ILT-2 and ILT-4 is more effective in myeloid cell reprogramming in the TME in 3D tumor spheroids	(3, 181–183)
CD24 (a do not eat me signal; a checkpoint for phagocytosis)	SIGLEC-10, SIGLEC-15; P-selectin, Integrin β1, NKG2D	Hematopoietic and non-hematopoietic cells constitutively express CD24; Cancer stem cells overexpress CD24; Ligands: NKG2D on NK and CD8+ T cells, SIGLECs are expressed on T, B, Macs, Monocytes and DC; P-selectin is expressed by activated platelets and endothelial cells; Integrin β-1 is expressed by cancer cells	CD24 blocker promotes cancer cell phagocytosis; Inhibits cancer cell metastasis, progression and chemo-resistance by inhibiting β1 integrin stability; Promotes NK cell and CD8+ T cell-mediated killing of cancer cells via NKG2D	IMM-47 (IgG1); (IgG4); ONC-781 (IgG4) specifically targets CD24 on cancer cells but not on normal cells	(184–186)

APC, Antigen presenting cells; ADCC, Antibody-dependent cell mediated cytotoxicity; B7-H5, B7 family homolog-5, another name for VISTA; CEACAM, Carcinoembryonic antigen-related cell adhesion molecule; CTLA-4, Cytotoxic T lymphocyte-associated Antigen-4; DC, dendritic cells; FGL-1, Fibrinogen-like protein-1; Gal, Galectin; LAG-3, Lymphocyte Activation Gene-3; HAVCR2, Human Hepatitis A virus cellular receptor-2; HMGB1, High mobility group protein B-1; ILT-2, 4, Immunoglobulin-like transcript 2, 4; LILRB, Leukocyte Immunoglobulin-like receptor subfamily B; LIRG1, Leucine-rich repeats and immunoglobulin-like domains 1; LSECTIN, Liver sinusoidal endothelial cell lectin; MDSC, Myeloid-derived suppressor cells; NKG2D, Natural Killer cell group 2 member D, an activating receptor expressing by NK cells and a subset of CD8+ T cells; PS, Phosphatidylserine; PSLG-1, P-selectin glycoprotein ligand-1; PVRL-2, Poliovirus receptor like-2/CD112/Nectin-2; PVRL-3, Poliovirus receptor like-3/CD113/Nectin-3; PVR, Poliovirus receptor/CD155; SIGLEC, Sialic acid-binding immunoglobulin-type lectins; TGIT, T-cell immunoglobulin and ITIM domain; TIM-3, T cell Immunoglobulin & mucin domain-3; VISG3/8, V-Set and Immunoglobulin domain containing 3/8; VISTA, V-domain immunoglobulin suppressor of T-cell activation.

**Figure 1:**
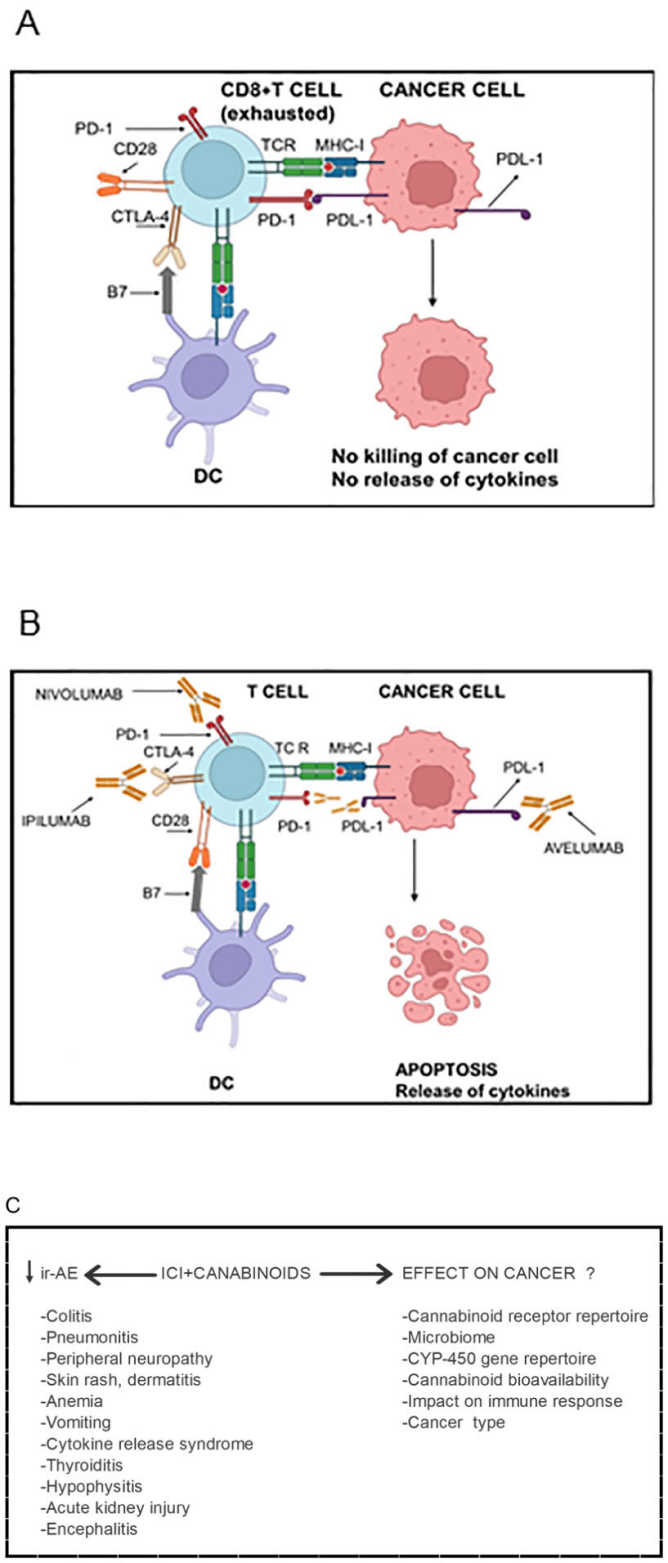
Cannabinoids’ impact on Immune checkpoint inhibitor (ICI) immunotherapy in cancer patients. **(A)** Activated T cells express higher levels of CTLA-4, which, compared with CD28, has higher affinity and avidity for B7 (CD80 and CD86) expressed by antigen-presenting cells. Consequently, CTLA-4 supersedes CD28 for binding with B7 molecules. CTLA-4 mediated inhibitory signals inactivate T cells rendering them “exhausted”. Due to chronic antigenic stimulation, T cells also express other inhibitory checkpoints such as PD-1. The PD-1 expressing T cells encounter PDL-1 expressed by cancer cells as well as by myeloid cells in the tumor microenvironment. The PD-1/PDL-1 interaction inhibits effector functions of both CD4+ and CD8+ T cells such as secretion of cytokines, presentation of antigens to cognate B cells and killing of cancer cells. Consequently, cancer cells evade anti-cancer immune immunity. **(B)** Immune checkpoint blocker Ipilumab binds CTLA-4 on T cells and inhibits its interaction with B7 molecules allowing the interaction of CD28 with B7 and hence T cell activation. Nivolumab binds PD and inhibits its interaction with PDL-1 liberating T cells from inhibitory signals mediated through the PD-1/PDL-1 interaction. The interaction can also be blocked Nivolumab which binds PDL-1 and blocks its interaction with PD-1. Being of IgG1 isotype, Avelumab can also kill PDL-1 expressing cancer cells as well as PDL-1-expressing myeloid cells in the tumor microenvironment through antibody-dependent cell-mediated cytotoxicity. The ICIs enable cancer-specific CD8+ T cells to kill cancer cells and release cytokines resulting in cancer regression. However, ICI immunotherapy results in several toxic side effects called immune-related adverse events (ir-AE). **(C)** The addition of cannabinoids to the ICIs attenuates severity of ir-AE and increases tolerability of ICIs. However, cannabinoids’ effects on the efficacy of the immunotherapy yet remain controversial. They depend upon several factors listed in the lower panel. Figure re-drawn after (1, 165).

The first ICI approved by FDA in 2011 for advanced melanoma patients was Ipilumab (6), a humanized mAb of IgG1 isotype that targets CTLA-4. CTLA-4 is a CD28 homolog that binds B7 (CD80 and CD86) molecules expressed constitutively on the surface of antigen presenting cells (APC) with much higher affinity and avidity than CD28, a constitutively expressed co-stimulatory molecule on T cells (**Figure 1**). CTLA-4 expression is intrinsically linked with T cell activation through T cell receptor (TCR), and upon this activation, CTLA-4 is rapidly mobilized from its intracellular vesicular stores to the cell surface. The expression peaks in 2-3 days following TCR engagement, and correlates with the strength of the TCR activation. It supersedes CD28 for binding with B7 molecules and thus limits/deprives T cells from CD28-induced co-stimulatory signaling via activation of PI3K and AKT (5). ICIs targeting CTLA-4 inhibit CTLA-4/B7 interactions allowing CD28/B7 interactions. This amplifies TCR-mediated T cell activation. As immunosuppressive Tregs constitutively express high levels of CTLA-4, CTLA-4-blocking antibodies (**Table 1**) also inhibit as well as eliminate Tregs through ADCC and ADCP. These antibodies are less effective in aged patients as they have reduced numbers of Tregs. Furthermore, anti-CTLA-4 antibodies reduce expression of CD80 and CD86 on interacting APC through trogocytosis promoting immunosuppression. Soon it was discovered that monotherapy with anti-CTLA-4 ICIs leads to compensatory expression of other inhibitory checkpoints such as PD-1 and others (see below) resulting in resistance to the ICI.

PD-1, as well as its ligand, PDL-1, is now the most widely targeted checkpoint in cancer patients. Several humanized mAb for targeting PD-1 have been developed. For example, Nivolumab, Pembrolizumab, Cemiplimab and Dostarlimab target PD-1 and inhibit their interaction with PDL-1 expressed on cancer cells as well as on non-cancer cells in the tumor microenvironment (TME). All these antibodies are of IgG4 isotype, lack FcR-mediated effector functions and are meant to inhibit PD-1 interaction with PDL-1. Interestingly, anti-PD-1 ICIs are more effective in cancer patients that express PDL-1 in the TME. Such cancer patients have anti-cancer immune responses and are said to have “hot” tumors. PD-1-specific ICIs are more effective than the CTLA-4-specific ones, and are comparatively less toxic. The PD-1/PDL-1 interaction attenuates TCR-mediated signaling through activating SH-2 domain-containing protein tyrosine phosphatase (SHP)-2, which dephosphorylates several key molecules implicated in TCR-mediated signaling. Furthermore, it also blocks CD28/B7-mediated co-stimulatory signals. Anti-CTLA-4 antibodies mainly lead to the expansion of PD-1+ ICOS+T-bet+ CD4+ T cells, whereas anti-PD-1 antibodies primarily result in the expansion of CD8+ T cells. PD-1 ligation on T cells promotes fatty acid oxidation (FAO) but attenuates glycolysis, whereas CTLA-4 ligation leads to attenuation of glycolysis without affecting FAO. The blockade of the two inhibitory checkpoints reverses the metabolic changes in exhausted T cells and reinvigorates them (5, 7). Furthermore, it decreases threshold of T cell activation permitting activation of low affinity TCR resulting in so called “epitope spreading”. The low affinity TCR-bearing T cells recognize neo-antigens expressed by cancer cells. Not surprisingly, combined immunotherapy with anti-CTLA-4 and anti-PD-1, due to their synergism, gives better responses in cancer patients. The synergism also results, at least in part, from the fact that anti-CTLA-4 antibodies target circulating T cells while anti-PD-1 antibodies target tumor infiltrated T cells. The combined immunotherapy also gives better responses in PDL-1 negative tumors. The PD-1 ligand/counter-receptor, PDL-1, has also been targeted by humanized mAbs of IgG1 such as Avelumab and Atezolizumab (**Table 1**). In addition to inhibiting PD-1/PDL-1 interaction through steric hindrance, anti-PDL-1 antibodies kill PDL-1-expressing cancer cells as well as non-cancer cells in the TME through ADCC and ADCP. As PDL-1-mediated signaling promotes aerobic glycolysis as the primary energy source, and promote cell proliferation through PIK-3/Akt/mTOR pathway, its blockage puts metabolic constraints in cancer cells (5). Not surprisingly, PDL-1 targeting ICIs show efficacy in only in PDL-1+ cancers (5, 7).

The pathophysiology of immune checkpoints is complex. Certain chemotherapeutic agents can induce expression of PDL-1 in cancer cells (8). In addition, oncogenic signals such as epidermal growth factor receptor (EGFR) activation, certain and TH1 cytokines such as IFN-γ and TNF-α also induce its expression in cancer cells as well as in non-cancer cells (such as fibroblasts and myeloid cells) in the TME. PDL-1 expressing macrophages inhibit infiltration of T cells in the TME (9). In contrast to TH1 cytokines, TH2 ones induce expression of PDL-2 on APC; not much is known about its significance in the checkpoint immunotherapy. Concerning, PD-1, persistent antigenic stimulation, IFN-α and -β and TLR stimulation induce expression of PD-1 in T cells and other immune cells such as macrophages, NK and B cells (10, 11). Other than being expressed in immune cells, CTLA-4 and PD-1 are also expressed in cancer cells. *In vitro*, anti-CTLA-4 antibody activates EGFR pathway in CTLA-4 expressing cancer cells, induces cell proliferation as well as the expression of PDL-1 in the cancer cells. Tumor-intrinsic PD-1 is also expressed in a subset of PDL-1^+^ cells in a broad range of cancer types; the two act in cis (when present on the same cancer cell) and suppress cancer cell proliferation *in vitro* in the absence of adaptive immunity. However, the interaction also makes the cancer cells resistant to the ICIs (12). In addition to their membrane-bound forms, the checkpoints such as CTLA-4, PD-1, PDL-1 and others are also expressed in soluble (s) forms (13, 14), which result either from their mRNA splice variants or from the shedding of their membrane-bound forms through proteolytic cleavage by matrix metalloproteases (MMP)-7, 9 and 13, as well as by the ADAM (a disintegrin and metalloproteinase) family member-10 and -17. The soluble forms bear implications for ICI immunotherapy. For example, sCTLA-4 binds B7 molecules (B7-1/CD80 and B7-2/CD86) on antigen presenting cells (APC) and prevents their interaction with the T cell co-stimulatory molecule CD28. In a murine model of cervical adenocarcinoma, sCTLA-4 was shown to attenuate CD8+ T cells and promote cancer progression (14). On the other hand, sPD-1 blocks PD-1/PDL-1 interactions (acts as an immune checkpoint blocker); higher plasma concentrations of sPD-1 in untreated anal and pancreatic ductal adenocarcinoma patients correlate with better prognosis (11). Unlike sPD-1, sPDL-1 can bind with PD-1 on immune cells and inactivate them; increased levels of sPDL-1 in cancer patients correlate with worse prognosis. It acts as a decoy for anti-PDL-1 antibodies necessitating higher doses of ICIs (11, 15).

It is noteworthy that PD-1 is a marker of cell activation, and only a subset of PD-1+ T cells are exhausted. The exhausted T cells also express other inhibitory immune checkpoints most notably LAG-3 and TIM-3. Accordingly, in addition to CTLA-4 and PD-1/PDL-1, humanized antibodies for these and several other checkpoints have been developed and are in various stages in clinical development. The checkpoints include LAG-3, TIM-3, TIGIT, ILT2/4, VISTA, NKG2A and CD24; see **Table 1** for their expression, ligands/counter receptors, blocking antibodies and potential mechanisms of action. Many studies have been undertaken to investigate dual targeting of PD-1/PDL-1 with LAG-3, TIM-3 or TIGIT with better and less toxic side effects as compared with the combination of PD-1/PDL-1 with CTLA-4 (16). Furthermore, antibodies have been developed that simultaneously target two different checkpoints. For example, Cadonilimab is a tetravalent bispecific antibody of IgG1 isotype that targets CTLA-4 and PD-1 simultaneously (17). It has been approved for advanced or relapsed cervical, gastric and gastro-esophageal junction cancers. In addition, Lomvastomig targets both PD-1 and TIM-3, and KN046 targets CTLA-4 and PDL-1 (16, 18). Studies in murine models have shown that simultaneous targeting of two or more different checkpoints is more effective in regressing cancers. However, they are also more toxic.

ICI immunotherapy has been used as a stand-alone and/or as an adjunctive therapy (in combination with chemotherapy) in a wide variety of cancers including melanoma, breast cancer, NSCLC, RCC, colorectal cancer (CRC), prostate, pancreatic and breast cancers as well as in hematological malignancies with variable response rates (RR). Depending upon the type of the cancer, the RR may vary between 20-50%. The responses have been in general better in advanced stage metastatic cancers. Unfortunately, only a small subset of patients responds to ICIs in each cancer type. Exact reasons for a low response are not known. It has been observed that tumors with higher mutational burden, microsatellite instability, inflamed stroma as well as increased infiltration of immune cells respond better to ICIs. Furthermore, altered signaling pathways and genetic polymorphism in checkpoint genes such as CTLA-4, PDCD (which encodes PD-1) and PDL-1 may play a role in variable responses to ICIs in cancer patients (19).

A major limitation of ICI immunotherapy is that it is accompanied by moderate to severe immune-related adverse events (ir-AE) including nausea, vomiting, dysphagia, skin rashes, cytokine release syndrome (CRS), dermatitis, colitis, hepatitis, hypophysitis, thyroiditis/hypothyroidism, myocarditis and neurological disease (**Figure 1**). The ir-AE are different from those caused by chemotherapy. They are likely to result from the above normal activation of T cells due to a lowered threshold of TCR activation. Consequently, there occurs activation of T cells with low affinity TCR that would otherwise not be activated. Furthermore, an early increase in clonal proliferation of PD-1+ CD21-low B cells was observed and correlated with ir-AE in cancer patients following anti-PD-1 and anti-CTLA-4 therapies (5, 20). It is noteworthy that mice deficient in CTLA-4, PD-1 or PD-L1 develop various lymphoproliferative disorders and autoimmune manifestations (21). Not surprisingly, in their clinical presentation, ir-AE resemble autoimmune diseases. They may be acute or chronic, and may persist even after cessation of the immunotherapy. In up to 1.23% of the patients, the ir-AE could be fatal (22, 23). These events often lead to discontinuation of ICI immunotherapy. Irony is that effectiveness of the ICI immunotherapy correlates with their toxicity. To counter these adverse events, and to increase tolerability of ICIs, steroidal anti-inflammatory drugs (SAID) such as prednisone, and non-steroidal anti-inflammatory drugs (NSAID) such as Ibuprofen, intravenous immunoglobulins (IVIG) and TNF-α inhibitors, etc., have been used (24–26). However, these medications were found to be associated with worse clinical outcomes in ICI therapies. More recently, researchers have resorted to using cannabinoids instead of SAIDS or NSAIDS to limit ir-AE and increase tolerability of ICIs.

## 3 Cannabinoids

Cannabinoids refer to 21-carbon terpenophenolic background compounds and their derivatives isolated from the plant Cannabis sativa (C. sativa), commonly known as Marijuana. They were named cannabinoids signifying their origin from the cannabis plant. The plant has been used for medicinal and recreational purposes since millennia. To date, more than 125 different cannabinoids have been identified in this plant. The cannabinoids from the cannabis plant are more specifically phytocannabinoids. Other cannabinoids include endocannabinoids, which are produced in our bodies and synthetic cannabinoids, which include various structurally diverse cannabimimetic compounds that are synthesized in laboratories. Other names used for phytocannabinoids are natural cannabinoids or exocannabinoids (27–29). Two most studied phytocannabinoids are Δ9-tetrahydrocannabinol (THC) and cannabidiol (CBD). They are derived from their precursor molecule, cannabigerolic acid (CBGA), in cannbis. About 95% of the cannabinoids in cannabis plants exist in their acid forms such as THC-acid (THCA), cannabidiolic acid (CBDA) and cannabichromenic acid (CBCA), which are synthesized from CBGA through the action of cannabinoid-specific synthases. The acidic forms protect cannabis plants acting as anti-oxidants, insecticides and microbicides. The acidic forms are non-enzymatically decarboxylated to yield neutral cannabinoids THC, CBD, CBC and CBG (from CBGA) when exposed to high temperatures, light and oxygen as well as prolonged storage (29, 30). It may be relevant to mention here that THCA is converted to THC upon smoking or vaping cannabis. Furthermore, CBD found as nano-emulsion in energy drinks is converted to psychoactive THC under acidic conditions of the stomach (31).

Relative to THC and CBD, CBC, CBG, cannabinol (CBN), cannabidivarin (CBDV), tetrahydrocannabivarin (THCV), hexahydrocannabinol (HHC) and Δ-8 tetrahydrocannabinol (Δ-8 THC) are also found in minor or trace amounts in cannabis (30, 32). Of these, CBN is an oxygenation product of THC; CBDV and THCV have propyl side chains instead of usual pentyl ones in CBD and THC from whom they are derived; HHC is a hydrogenated derivative of THC, and Δ-8 THC is an isomer of THC with a double bond beginning at position 8, instead of position 9. In addition, Abnormal-CBD (Ab-CBD) is a synthetic regio-isomer of CBD, and is found as an impurity when cannabinoids are synthesized in laboratory (33). Of the cannabinoids, THC exerts its psychoactive effects mainly through its ability to activate the first discovered cannabinoid receptor (CB1), and is also responsible for the psychoactive effects of cannabis. Some other cannabinoids such as HHC, Δ-8 THC and CBN also have psychoactive effects, however they are less potent than THC in equivalent doses. On the other hand, CBD and CBG lack psychoactive effects. In fact, they counter some of THC’s psychoactive effects (30). *In vitro*, Δ-8 THC is synthesized from CBD, and is illegally marketed as it is not specifically prohibited in the 2018 Farm Bill in the USA (31).

The genus cannabis contains three species, C. sativa, C. indica and C. ruderalis representing low, high and intermediate levels of THC-containing species, respectively. Most strains or cultivars currently grown are hybrid species. They are grown to obtain fiber, seeds (rich in unsaturated fatty acids for edible oil) or for recreational/medicinal purposes. Those used for obtaining fiber and food are legally required to contain < 0.03% THC (by dry weight) and are called ‘hemp’; others (drug type) contain as much as 30% THC. Cannabis strains or cultivars are selectively bred for specific traits, for example for their THC, CBD, CBC, unsaturated oils or fiber contents (34, 35). It is noteworthy that in addition to cannabinoids, cannabis plants also contain a diverse array of many other compounds including terpenoids (e.g., β-caryophyllene), flavonoids (e.g., quercetin), phenols and other phytochemicals. Current trend is to define cannabis cultivars based upon their biochemical constituents into “chemovars” (34).

Several cannabinoid preparations are currently available commercially. Two synthetic THC analogs (Nabilone and Dronabinol) have been approved by FDA for chemotherapy-induced nausea, anorexia and vomiting in cancer patients (36). Dronabinol is also used in people living with HIV (PLWH) for improving appetite and preventing weight loss. The THC preparations are also used for other therapeutic applications including glaucoma, migraine, headache, anorexia, spasticity, anxiety, and pain. The second major phytocannabinoid, CBD, is well known for its anti-convulsive and neuroprotective effects. Nabiximols (Sativex) is a cannabis extract containing THC and CBD in 1:1 molar ratio and is used as buccal spray for alleviating neuropathic pain, multiple sclerosis spasticity and overactive bladder. On the other hand, Epidiolex, contains purified CBD from cannabis plants and is approved for controlling seizures in therapy-resistant childhood epilepsy as well as in a rare and severe form of epilepsy, namely Lennox-Gastaut syndrome or Dravet syndrome. CBD has great therapeutic potential due to its antipsychotic, antidepressant, anxiolytic, anti-inflammatory and analgesic effects (37). It is non psychoactive, has little potential for abuse and is often the preferred choice for cannabis-based therapies. Another preparation, Spectrum Yellow Oil contains CBD (20 mg), THC (0.9 mg) and CBC (1.1 mg) per ml and a candidate for treating neuroinflammatory conditions (38).

**Figure 2:**
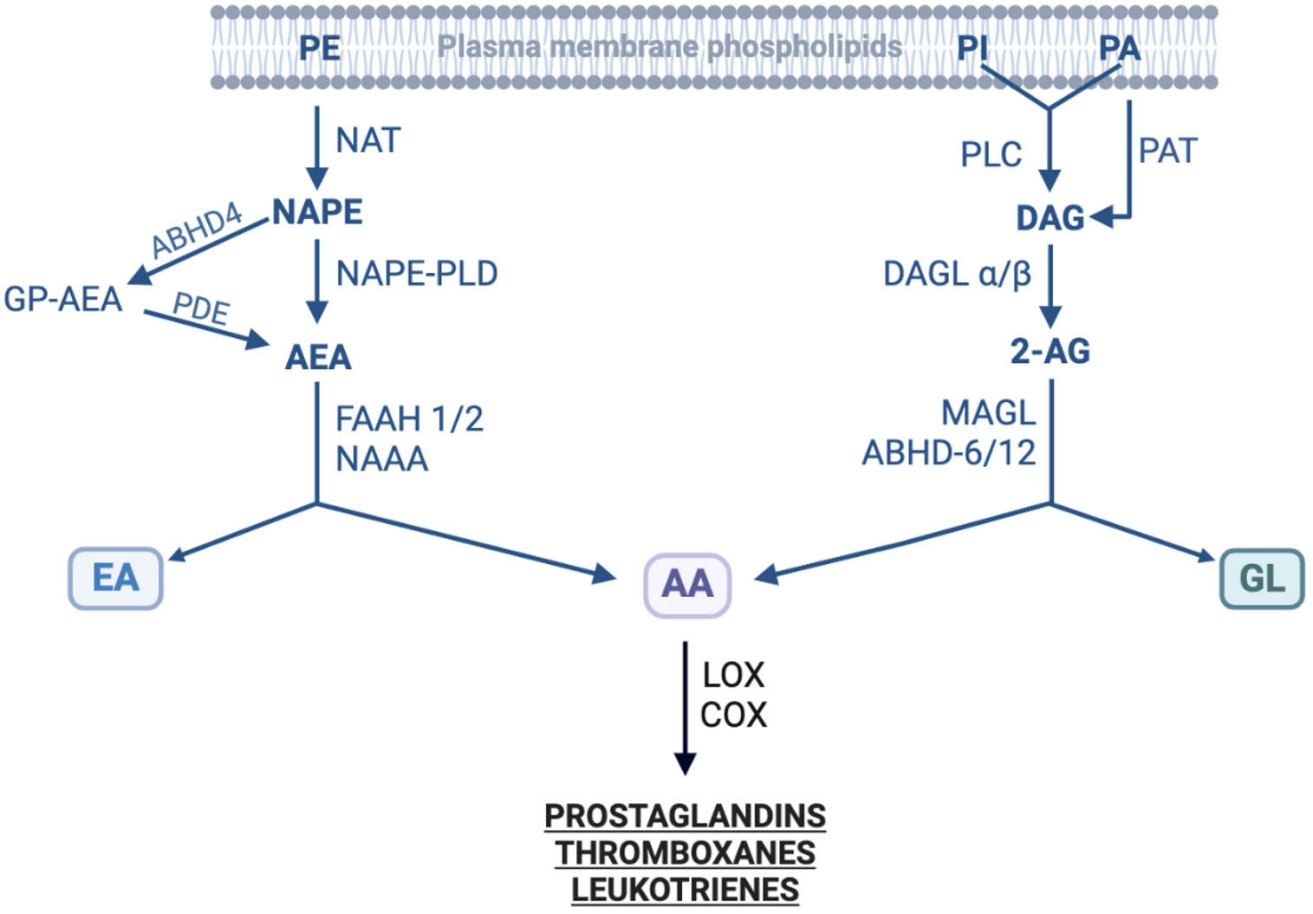
Enzymes involved in the biosynthesis and degradation of endocannabinoids. The figure shows enzymes involved in the synthesis of two families of endocannabinoids from their precursors as well as in their metabolic degradation. 2-AG, 2-Arachidonoylglycerol; AA, Arachidonic acid; ABHD, α/β hydrolase domain-containing (or N-Acylphospholipase B); AEA, N-Acylethanolamide; COX, Cyclooxygenase; DAG, Diacylglycerol; DAGL, Diacylglycerol lipase; EA, Ethanolamine; FAAH, Fatty acid amide hydrolase; GL, Glycerol; GP-AEA, Glycerophosphate-AEA; LOX, Lipoxygenase; MAGL, Monoacylglycerol lipase; NAAA, N-Acylethanolamide acid amidase; NAPE, N-Acylphosphatidyl ethanolamine; NAPE-PLD, NAPE-specific phospholipase D; NAT, N-Acyltransferase; PA, Phosphatidic acid; PAH, Phosphatidic acid phosphohydrolase; PDE, Phosphoglycerodiesterase-E; PI, Phosphoinositide; PE, Phosphatidylethanolamine; PLC, Phospholipase C; PLD, Phospholipase D. Modified from (166–169); created with BioRender.

Studies on THC’s psychoactive effects resulted in the identification of the first cannabinoid receptor (CB1) in human and rat cells in 1988 (39). Search for endogenous ligands of CB1 led to the discovery of endocannabinoids, the cannabinoids produced in our bodies. Two most studied endocannabinoids include N-arachidonoylethanolamine (AEA; also known as anandamide meaning bliss in Sanskrit) and 2-arachidonoylglycerol (2-AG). They bind and activate several canonical and non-canonical cannabinoid receptors and exert their biological effects (**Table 2** and discussed below). A variety of tissues in the body including brain, muscle, fatty tissue, spleen, liver and pancreas as well as immune and non-immune cells produce small quantities of endocannabinoids, and can be measured in the circulation (40). Endocannabinoids are synthesized in the body from arachidonic acid (AA) and phospholipids, important constituents of the plasma membrane (**Figure 2**). The endocannabinoid system (ECS) maintains physiological homeostasis in the body by interacting with several neurotransmitters (acetylcholine, norepinephrine, glutamate, γ-aminobutyric acid, dopamine, serotonin and endorphins) and immune system. The system, in addition to endocannabinoids, comprises enzymes that synthesize endocannabinoids, enzymes that degrade endocannabinoids in the body as well as several receptors through which endocannabinoids mediate their biological effects (41). In addition to two main endocannabinoids, AEA and 2-AG, a large number of cannabinoid-like lipid mediators have been identified in the body that are related to the N-acylethanolamines (NAE) or Acylglycerol (AG) families. They include 2-arachidonyl glyceryl ether (Noladin ether), O-arachidonyl ethanolamine (Virodhamine), N-arachidonyl dopamine (NADA), N-palmitoyl ethanolamide (PEA), oleoylethanolamine (OEA), 1-palmitoylglycerol (1-PG) and 2-palmitoylglycerol (2-PG), etc. Endocannabinoids have very short half-life, are rapidly taken up by cells through transporters and are rapidly degraded. Two enzymes, fatty acid amide hydrolase (FAAH) and monoacylglycerol lipase (MAGL) hydrolyze and degrade AEA and 2-AG in post-synaptic and pre-synaptic neurons, respectively. FAAH is the rate limiting enzyme for degrading and liberating AA from AEA and related endocannabinoids of the NAE family. The inhibition of FAAH results in increased concentrations of AEA and suppresses inflammation in a mouse model of colitis (42). Several FAAH selective inhibitors have been developed (43). In addition, MAGL-specific inhibitors have also been developed; they degrade 2-AG and other endocannabinoids of the AG family (44). Importantly, inhibitors of the endocannabinoid degrading enzymes are being researched for potential clinical applications. Interestingly, phytocannabinoids can also regulate endocannabinoids in the body. Being lipophilic substances, phytocannabinoids permeate through plasma membrane and are chaperoned by intracellular fatty acid binding proteins (FABP)-3, 5 and 6. The FABP transport them to FAAH for degradation. The phytocannabinoids, THC and CBD, compete with endocannabinoids for binding with the FABP, and decrease FAAH-mediated degradation of AEA and related endocannabinoids. Recently, CBC was shown to inhibit MAGL and the cellular re-uptake of endocannabinoids (38). Normally, the plasma concentrations of AEA and its congeners relative to 2-AG and its congeners are higher in adult humans but the balance may change in disease conditions (45).

**Table 2:** Cannabinoids receptors, their location and ligands, signaling mechanism and effect on cancer.

Receptor	Ligands	Receptor location	Signaling mechanism	Relevance to ICIs and cancer	References
CB-1	THC and AEA (partial agonists), HHC and CBN are weak agonists, Allosteric inverse agonists: CBD, CBG THCV antagonist 2-AG full agonist	Peripheral afferent and somatosensory neurons in CNS; microglia, astrocytes and oligodendrocytes; many types of non-neuronal body cells and in cancer cells	Gαi/o, Gαs; activation of A type K+ channel; inhibition of Ca++ current; β-arrestin	Context and cancer dependent pro-and anti-cancer effects; analgesia, psychosis, euphoria, addiction, anti-nociception, memory formation, orexia and synaptic plasticity	(30, 54, 56–59, 102)
CB-2	THC and AEA (partial agonists); HHC, CBG, CBN, AEA and CBC are weak agonists; 2-AG is a full agonist	Immunocytes, microglia, certain non-immune cells such as synoviocytes; certain neurons in brain	Gαi/o, Gαs, Gα11/q, Gα12/13; β-arrestin	Context and cancer dependent pro-and anti-cancer effects; reduced neuronal excitability, anti-emetic, anti-epileptic, anxiolytic, anti-inflammatory, immunosuppressive	(30, 54, 56, 63, 66, 67, 102, 122)
GPR-55 (putative 3^rd^ cannabinoid receptor or CB3)	Agonists: LPI, Curcumin, THC, Ab-CBD; Antagonists: CBG and CBD	Cerebellum, GIT, bone, immune cells (NK and monocytes)	Gα11/q, Gα12/13; β-arrestin	LPI: cancer progression and inflammation; Ab-CBD and THC: anti-convulsive effects; CBG and CBD: anti-cancer, anti-inflammatory effects; Curcumin: glucose homeostasis	(71, 77–79, 102)
GPR-18	THC, AEA, NAGly, Ab-CBD, Viberalin, RVD2	Plasma membrane and cytosol, immune and non-immune cells in various tissues	Gαi/o, Gαq, β-arrestin	Inflammation resolution, inhibition of apoptosis, pain relief, a marker for tumor infiltrating lymphocytes, metastasis	(80, 81)
VDAC-1	Antagonists: CBD, CBG	OMM, plasma membrane	Metabolism regulation, Mitochondrial fragmentation, ↑ROS, apoptosis	Anti-tumor effects	(96, 97)
TRPV-1	CBD, CBG, CBDV, Capsaicin and other vanilloids, heat (>43°C) and protons	Peripheral sensory Neurons, CNS, VEC, heart, lungs; plasma membrane, ER	Ca++ influx, ER stress, cell survival, proliferation and metastasis; apoptosis upon inhibition	Analgesia, Nociception to heat, apoptosis, anti-cancer effects	(78, 83, 187)
TRPV-2	High temperature, THC, CBD, CBG, THCV, Heat, NPro, NTyr	Sensory neurons, immune cells, many organs; plasma membrane, ER	Ca++ influx, cell survival, proliferation, metastasis; inhibition leads to apoptosis	Nociception, analgesia, metastasis	(83, 84, 187)
TRPV-3	Camphor, carvacrol, CBD, CBDV, CBG, THC; antagonists: NVal	Sensory peripheral neurons in DRG and trigeminal ganglia, testis, skin, oral epithelium	Ca++ influx, release of histamine	Allodynia to innocuously warm and hot temperatures, itching, cancer pain; both pro- and anti-cancer effects	(83, 89)
TRPV-4	AEA, 2-AG, CBG, CBN, THCV, THC, NTrp, NTyr, hypo-osmotic, mecha-nical and thermal stimuli (25-34°C) omega-3 PUFA, AA, H_2_O_2_, PGE2	Afferent sensory neurons in DRG and trigeminal ganglia, EC, OB, OC, heart, liver, skin, epithelia, kidney, blood vessels, immune cells	Mechanosensitive Ca++ channel	Differentiation of neutrophils, macrophage function, nociception, analgesia upon desensitization, stiffens tumor microenvironment; anti-cancer progression	(90, 91)
TRPA-1	Bradykinin, cold (<17°C), ROS, RNS, RCS, isothioacyanates found in mustard, garlic, clove and onion (eugenol), LPS, AEA, AA, CBD, CBCN, CBG, THC and CBC	Peripheral sensory neurons, non-neuronal cell such as monocytes, macrophages, T and B cells, cancer cells, VEC	Agonist-induced Ca++ influx	Nociception, inflammatory and neuropathic pain, hyperalgesia and allodynia; redox adaptation; cancer pain, angiogenesis	(83, 94, 102, 187)
TRPM-8	LPI, cold (<25°C), methanol, mechanical pressure, testosterone; Antagonists: CBD, CBG, CBDV, AEA, NADA	Neuronal and non-neuronal tissues	A nonselective cation channel; activation of PLC, PKC and RAS/ERK pathway	Cell proliferation, EMT, metastasis, cold hyperaglesia	(92, 93)
PPAR-α	CBD, THC, AEA, 2-AG, OEA, PEA, UFA, LTB4, 1-PG, 2-PG and Fibrates	Liver, heart, skeletal muscles, small intestine	Gene transcription	Anti-cancer, anti-nociceptive	(98, 101)
PPAR-γ	Endogenous: UFA, AA, OA, phospholipids, lysophosphatidic acid; Cannabinoids: CBG, CBD, THC; Synthetic ligands: Glitazones	Adipocytes	Gene transcription; ↑Autophagic flux and apoptosis	Cancer type dependent anti- and pro-cancer effects CBD reduces neurodegeneration and stimulates neurogenesis	(98–100, 102)
5HT-1A 5HT-1B 5HT-2A 5HT-7	Agonists: THC, AEA CBD is inverse agonist	Central and peripheral nervous system	Gαi/o, inhibit AC Gαq/11	Analgesic, anti-emetic, anxiolytic and anti-epileptic	(104, 105)
GlyR-α3	Strychnine (high affinity antagonist), CBD, THC	A widely distributed inhibitory channel in CNS	Ligand gated Cl- channel	Analgesia Hyperekplexia	(60)
NMDAR	Glutamate; co-agonists: glycine and D-serine; THC, CBD	Expressed on microglia, and post-synaptic region of glutamatergic neurons; non-neural cells such as epithelial cells and immune cells	Excitatory ionotropic glutamate receptor, permeates cations; cannabinoids attenuate neural excitotoxicty and prevent neuronal death;	Psychiatric disorders, neurodegenerative diseases; cannabinoids protect from epileptic seizures and neuronal cell death and exert anti-cancer effects	(109, 113, 115)

Ab-CBD, Abnormal CBD (a positional isomer of CBD); AC, Adenylate cyclase; CSC, Cancer stem-like cells; DRG, Dorsal root ganglia; EMT, Epithelial to mesenchymal transition; ENT-1, Equilibrative nucleoside transporter-1; GPR, G-protein coupled receptor; LPI, Lysophosphatidylinositol; 5-HTR, 5-Hydroxytryptamine receptor; CBD, Cannabidiol; CBG, Cannabigerol; P, in brackets indicates partial agonist; TRPA-1, transient receptor potential ankyrin 1; PPAR, Peroxisome Proliferator-Activated Receptor; 2-OG, 2-Oleoylglycerol; UFA, Unsaturated fatty acids; TRPM-8, Transient Receptor Potential Melastatin-8; NAGly, N-arachidonyl glycine (a metabolite of AEA); NVal, N-Acylvaline; PUFA, Polyunsaturated fatty acids; ReV-D2, Resolvin-D2; VEC, Vascular endothelial cells; OB, Osteoblasts; OC, Osteoclasts; EC, Endothelial cells; CBCN, Cannabichromene; NGly, N Acyl glycine; NSer, N Acyl serine; RNS, Reactive nitrogen species; RCS, Reactive carbonyl species; ER, Endoplasmic reticulum; GlyR, Glycine receptors; HHC, Hexahydrocannabinol.

As discussed above, phytocannabinoids indirectly increase endocannabinoid tone in humans (46). However, it has to be noted that there is an intricate cross talk between endocannabinoid and prostanoid systems. The hydrolysis of endocannabinoids yields AA, a substrate for cyclooxygenase (COX) enzymes. The enzymes also metabolize EAE and 2-AG into prostaglandin-ethanolamides (PG-EA) and prostaglandin-glycerol esters (PG-G), respectively. The COX-generated metabolites such as PGD-2, PGE2, PGI-2, PGF2a-EA, and PGE2-G exert pro-inflammatory and pro-algesic effects countering some of cannabinoids’ biological effects (47). It follows that concurrent targeting of COX enzymes or PG synthases would augment beneficial effects of phytocannabinoids, FAAH and MAGL inhibitors concerning nociception and inflammation.

Typically, endocannabinoids are not produced constitutively in the body. Immune cells such as T and B cells, platelets and macrophages produce and release them upon activation (40). In the brain, endocannabinoids are produced on demand when certain post-synaptic receptors such as glutamate or acetylcholine are activated (28). The released endocannabinoids then act on pre-synaptic neurons in a retrograde fashion and generally inhibit the release of neurotransmitters such as dopamine, gamma-amino butyric acid, glutamate, serotonin, opioids and noradrenaline, etc. In addition, endocannabinoids are tonically produced by hippocampal neurons in response to hunger (28).

## 4 Pharmacokinetics of cannabinoids

Cannabinoids are mainly consumed orally, inhaled (smoked or vaped) or used as oromucosal (sublingual or inside cheeks) spray (48). The bioavailability of the consumed cannabinoids varies greatly depending upon their mode of consumption. When inhaled, cannabinoids enter blood stream through lungs within a few minutes. The bioavailability of THC and CBD via this route is 10-35% and 11-45%, respectively. Through vaping, cannabinoids can be inhaled without burning. During vaping, volatile cannabinoids are vaporized and inhaled when hot air passes through cannabis. Vaporization, like smoking, leads to higher availability of cannabinoids. Users can experience psychotropic effects within 15-30 minutes; the effects lasting for 2-3 hours. However, their bioavailability upon ingestion is only 4-10% for THC and 6% for CBD (49). In most of the experimental studies, cannabinoids are administered orally and no assessment is made as to the plasmatic concentrations of these cannabinoids or their metabolites in the patients. Being highly lipophilic, cannabinoids accumulate in adipose tissues of the body and are released over prolonged periods of time. For this reason, chronic users of cannabinoids may experience less severe withdrawal symptoms upon their cessation (48).

Drug metabolizing enzymes, Cytochrome P 450 (CYP450) and uridine diphosphate (UDP)-glucuronosyltransferases (UGT) are implicated in the metabolism of drugs and xenobiotics including cannabinoids (50, 51). They are mainly expressed in the mitochondria and endoplasmic reticulum of the cells in the liver and to a lesser extent in the intestines, lungs and kidneys. CYP450 are a superfamily of 57 isozymes that metabolize drugs through phase I reactions (oxidation, reduction and hydrolysis); of these, three subfamilies CYP1, 2 and 3 playing a major role in humans. UGT metabolize drugs and/or their phase I metabolites mainly through glucuronidation. The metabolic pathways render drugs more hydrophilic for clearance through hepatobiliary and renal excretory systems. The major psychoactive cannabinoid THC is mainly metabolized by CYP2C9 and CYP2C19 to 11-OH-THC (as psychoactive as THC) and then oxidized to inactive 11-COOH-THC, which is further converted to 11-COOH-THC-glucuronide by UGT1A and UGT1A3, and excreted (51, 52). The other major non-psychoactive cannabinoid CBD is mainly hydroxylated at position 7 by CYP2C9 and CYP2C19 into 7-OH-CBD, which is biologically active like CBD. On the other hand, CYP3A4 hydroxylates CBD at positions other than 7, and converts it into inactive metabolites; chemical inhibition of the CYP3 increases 7-OH-CBD (53). Rifampicin, a CYP3A4-inducing antibiotic decreases concentrations of CBD and 7-OH-CBD whereas Ketoconazole, a CYP3A-inhibiting fungicide, increases CBD and 7-OH-CBD in the circulation. The 7-OH-CBD metabolites are further metabolized to inactive 7-COOH-CBD and are excreted from the body. Un-metabolized CBD is also excreted from the body. The CBD metabolism involves very minor contributions from UGT enzymes. A fraction of it as well as of its metabolites are glucuronidated by UGT1A9 and UGT2B7 and are eliminated from the body.

## 5 Pharmacodynamics of cannabinoids

Cannabinoids exert their biological effects through canonical and non-canonical cannabinoid receptors (54) (**Table 2**). Their canonical receptors include CB1 and CB2, whereas non-canonical ones include G-protein coupled receptor (GPR)-55, GPR-18, different ion channels and peroxisome proliferator-activated receptors (PPARs). Through these receptors, cannabinoids activate multiple cell signaling pathways. They are discussed below:

### 5.1 CB1 (or CNR1)

This receptor is expressed mainly on the terminals of central and peripheral neurons in the in the brain and spinal cord. In particular, it is highly expressed in the hippocampus, hypothalamus, prefrontal cortex, nucleus accumbens, and cerebellum as well in emetic centers in the brainstem (55). It is expressed in pre-synaptic GABAergic, glutamatergic, serotoninergic, noradrenergic and cholinergic neurons, where it attenuates excitatory and inhibitory synaptic transmission. THC and its derivative HHC act as partial agonists for CB1. CB1 agonists provide anti-nociceptive effects and reduce pain (56). The cannabinoid action through CB1 is mainly responsible for addictive, euphorigenic and psychoactive effects of THC and cannabis. Outside the CNS, CB1 is also expressed in peripheral tissues such as gastro-intestinal tract, skeletal muscles, cardiovascular system, liver, skin and reproductive system. Upon ligand binding, CB1 couples with Gαi/o and inhibits adenylate cyclase, production of cAMP, inhibition of PKA and activation of MAPK through arrestin-β1. Interestingly, CB1 is also expressed on the mitochondrial outer membrane. It regulates a broad range of physiological processes in the body including neurotransmission; motor functions, sleep, emotions, memory, fear and reward, anxiety, pain, appetite and food intake, energy balance, secretion of gastric acids and fluids, oxidative stress, mitochondrial dynamics and cell death (57). Over-expression of this cannabinoid receptor has been detected in several types of human cancers, often as heteromers with other GPR (see below), and has been implicated in both cancer progression as well as in cancer inhibition depending upon the type of the cancer, nature of the heteromer as well as cellular context (58). Unlike THC, CBD acts as a non-competitive negative allosteric modulator of CB1, explaining its ability to counterbalance the psychoactive effects of THC (59). When CBD is present in adequate proportion (i.e. in balanced formulations of 1:1 molar ratio with THC) it can also attenuate the rewarding and anxiogenic effects of THC. CB1 dysregulation plays a negative role in neurological and psychiatric disorders, epilepsy, glaucoma, addiction, anxiety, and multiple sclerosis spasticity. These negative effects are ameliorated to a variable extent by biological effects of CBD though CB1 and other non-canonical cannabinoid receptors (60, 61). A wide variety of potent synthetic CB1 agonists (such as AM-1235, JWH-007 and HU-210) have been developed for research purpose. However, they are widely abused for recreational purpose and traded as illicit drugs. A synthetic CB1 antagonist, Rimonabant, was marketed as anti-obesity drug but was withdrawn from the market due to its suicidal ideation (62).

### 5.2 CB2 (or CNR2)

Unlike CB1, CB2 is expressed mainly in the peripheral tissues such as immune cells as well as in the brain on microglia, astrocytes and some neuronal cells in the brain. In addition to regulating the functional activities of immune cells, CB2 is implicated in the regulation of neural excitability, inflammation and nociception (63, 64). The anti-nociceptive and pain-relieving effects of CB2 agonists are free from the euphorogenic and addictive effects of CB1 agonists. THC acts as a partial agonist for CB2, whereas CBD has little or no affinity for this receptor. CB2 couples promiscuously with Gαi/o, Gαs, and other Gα proteins such as Gα11/q, Gα12/13. In primary human leukocytes, synthetic CB2 agonist HU308 was shown to couple concurrently with Gαs and Gαi, induce IL-6 and IL-10 production without any effect on cell numbers (65). It was also shown to activate p38 and exert growth inhibitory effect on cancer cells (65). Cannabis was shown to inhibit Jak-STAT-mediated signaling in immune cells mainly through CB2 (37). While CB2 agonists may promote tumor progression via their immunosuppressive effects, the receptor has also been demonstrated to promote cancer progression and metastasis through forming heteromers with epidermal growth factor receptor-2 (HER2) and CXCR4 (66, 67).

### 5.3 GPR-55

Dubbed as putative 3^rd^ cannabinoid receptor, GPR-55 bears sequence homology with CB1 and CB2 and forms heteromers with CB1 and CB2 (68, 69). Upon ligand binding, it couples with Gα11/q and Gα12/13. Lysophosphatidylinositol (LPI) and acylethanolamides act as its natural endogenous ligands (70). Its exogenous ligands include curcumin (an active ingredient of turmeric), Ab-CBD and THC; they inhibit proliferation of cancer cells (71, 72). Curcumin, via GPR-55, exerts antidiabetic effects. GPR-55 agonists antagonize cannabinoids’ CB1 and CB2-mediated effects and hold promise for substance abuse disorders (73). GPR-55 KO mice exhibit deficits in motor coordination and thermal sensitivity. Acting as antagonists of GPR-55, CBG and CBD antagonize the excitatory effects of LPI at hippocampal neurons and prevents seizures (74). The receptor also regulates nociception (mechanical hyperalgesia), production of pro-inflammatory mediators, bone metabolism, cardiovascular function and metabolism (57, 72, 75). GPR-55 is also expressed at higher levels in human NK cells, monocytes and microglia where it promotes production of pro-inflammatory cytokines and inflammation. Its antagonists exert anti-neuroinflammatory effects (76). CBG and CBD, acting as antagonists, inhibit GPR-55-mediated inflammation (77). Also, through antagonizing the cancer cell proliferating effects of LPI, they inhibit cancer progression (78). GPR-55 is overexpressed in many types of cancers and the expression correlates with cancer progression. The concentrations of its endogenous ligand, LPI, also increase in cancer patients (79).

### 5.4 GPR-18

It is activated by THC and Abn-CBD. Resolvin D2 (RvD2; a pro-resolving mediator that resolves inflammation) acts as its endogenous agonist and helps in inflammation resolution (80). It couples with Gαi/o and Gαq. GPR-18 agonists reduce inflammatory and neuropathic pain and ameliorate neurodegenerative diseases. However, they also inhibit apoptosis, promote cell proliferation and metastasis. The GPR also acts as a prognostic marker and indicates infiltration of B and cytotoxic T lymphocytes in several types of cancers (81).

### 5.5 Ion channels

Several ion channels act as non-canonical cannabinoid receptors; they include Transient Receptor Potential (TRP) channels and Voltage-dependent Anion Channel (VDAC)-1. TRP channels, as homo- or hetero-tetramers with six transmembrane helices in each subunit, are integral transmembrane proteins that form aqueous pores for permeation of cations (74). They play an essential role in cell’s functioning. They occur in the plasma membrane of afferent neurons in the dorsal root, Trigeminal and other somatosensory neurons as well as in several other cell types in different tissues in the body. Based upon their amino acid sequences, TRP are classified into seven subfamilies of which Vanilloid (TRPV), Ankyrin (TRPA) and Melastatin (TRPM) subfamilies are important in the context of cannabinoids (82). Several members of these TRP subfamilies act as ionotropic receptors for various cannabinoids (83). Interestingly, CB1 and CB2 co-localize with some TRP such as TRPV-1 in sensory and brain neurons. Cannabinoids desensitize TRP channels and produce analgesic effects. Under physiological conditions in non-neuronal cells, TRP activation may lead to cell proliferation, differentiation and apoptosis (**Table 2**). However, cancer cells overexpress or express abnormal TRP variants that result in altered ion transport and increased cell proliferation, migration, angiogenesis, metastasis and resistance to chemotherapy and radiation (84, 85). TRP are polymodal as each one can be activated by a diverse array of agonists. Their ligands include various mechanical, chemical and thermal stimuli, ROS and osmotic pressure, among others. Upon activation, TRP transduce signals as electrical currents in neuronal and non-neuronal cells (83).

#### 5.5.1 TRPV1 or Capsaicin receptor

It is activated by Capsaicin (an active ingredient found in red chillies), CBD, CBDV and CBG. The channel is also activated by low pH and temperature extremes. Expressed in peripheral nervous system and brain on nociceptive neurons, it is implicated in peripheral nociception, thermoregulation and synaptic plasticity (83). Agonists provide relief in neuropathic and inflammatory pain through desensitization of this ion channel. Capsaicin and resiniferatoxin (from Euphorbia resinifera plant) activate TRPV-1 eliciting strong burning sensation, then desensitize the channel providing an analgesic effect. TRPV-1 is overexpressed in a wide variety of cancers, and its expression correlates with poor prognosis; however, TRPV-1 activation suppresses development of gastric cancer (84). CBD exerts its anti-hyperalgesic effects through the desensitization of TRPV-1 at peripheral and spinal levels. Through TRPV-1, CBD induces Ca++ influx, ER stress and apoptosis in breast cancer cells (86). TRPV1 is sensitized by PGE2; the sensitization decreases activation threshold by its agonists (87).

#### 5.5.2 TRPV2

It is activated by high temperature, CBD, THC, THCV and Nabilone (a synthetic analog of THC) (83). Through the desensitization of TRPV-2, cannabinoids provide relief from heat-induced hyperalgesia. This is the only TRP for which THC acts as an agonist (88). The channel is overexpressed in a variety of cancers such as breast cancer, prostate cancer and others. Its expression in these cancers indicates poor prognosis. In glioblastoma, however, a loss of this TRP results in increased proliferation and resistance to FasL-mediated apoptosis (84).

#### 5.5.3 TRPV-3

This TRP is expressed in the brain, terminal ganglia, keratinocytes, oral epithelia and testis, etc. It causes pain and itchiness from otherwise innocuous warm temperatures (36-39°C) (83, 89). CBD, THCV, CBG and CBGV activate the channel. In addition to cooling agents, camphor and carvacrol, also activate this channel. TRPV-3 has oncogenic and metastatic potential and is overexpressed in certain cancers such as melanoma, breast cancer, and lung cancer (84).

#### 5.5.4 TRPV4

It has much wider distribution in the body in addition to brain and peripheral nerves. It is implicated in nociception in response to mechanical and osmotic stimuli (83). Its activators include CBD and THC analogs with prolyl side chain (CBDV and THCV). Interestingly, CBC was found to reduce TRPV-4 expression in the inflamed small intestine of mice. The TRP is overexpressed in several types of cancers including cancers of the breast, lung, liver, pancreas, stomach and colorectum. It has been implicated in matrix stiffness in the tumor microenvironment, which promotes cancer metabolism and metastasis (90). Modulating TRPV-4 activity in cancers that overexpress it not only inhibits cell proliferation but also enhances the efficacy of chemotherapy and radiotherapy while reducing cancer-associated pain. However, TRPV-4 functioning is necessary for host response to Mycobacteria; TRPV-4 KO mice show compromised migration and bactericidal activities of macrophages and neutrophils (91).

#### 5.5.5 TRPM-8 or cold receptor

This is a TRP of the melastatin (M) subfamily member 8, it is a tetramer of four identical subunits (92). It is expressed in a subset of C-type unmyelinated afferents located in the dorsal root and trigeminal ganglia as well as in non-neuronal tissues such as breast, prostate, lung and skin etc. It is activated by cold (below 27°C) and cooling compounds such as menthol, icilin (a synthetic menthol that is 200 times more potent than menthol) and euclyptol (an oil produced by the Eucalyptus spp.). TRPM-8 also plays a role in regulating vascular tone. Menthol and euclyptol increase blood flow to the area where they are applied. In the CNS, Nerve Growth Factor (NGF) induces expression of TRPM-8 which promotes neurite outgrowth and could mediate excitotoxic neuron death from noxious stimuli. Testosterone and estradiol act as agonists as well as regulate the expression of TRPM-8. The TRP is overexpressed in several types of cancers such as prostate, breast, lung and others. It promotes epithelial to mesenchymal transition (EMT), cell proliferation, metastasis and resistance to chemotherapy and irradiation in these cancers. It was demonstrated that in androgen-insensitive prostate cancer cells, TRPM-8 reduces cell proliferation. The TRPM-8-androgen interaction also explains neuronal effects of the sex hormone such as male aggressiveness (93). Most phytocannabinoids including THC and CBD act as TRPM-8 antagonists. They inhibit androgen-induced invasion and proliferation of prostate cancer cells. They also increase cytotoxic activity of chemotherapy and downregulate expression of PDL-1 on cancer cells (92). Interestingly, agonists such as menthol could also induce cancer cell death by inducing Ca++ influx, ROS production and apoptosis. The expression levels of TRPM-8 vary with stage of the cancer, which may affect the cancer’s response to the channel modulators.

#### 5.5.6 TRPA-1

It is a TRP of the Ankyrin family; it is expressed on a subset of sensory neurons. It is activated by bradykinin, mechanical pressure, low temperature (<17°C), eicosanoids of the arachidonate 12-lipoxygenase pathway, pungent compounds (allyl isothiocyanates) found in mustard, garlic and onion (83). It is co-expressed with TRPV-1 on peripheral sensory neurons and is important for nociception. Implicated in inflammatory and neuropathic pain, its most potent ligands are CBC, CBD, CBN and ACEA (arachidonyl 2-chloroethylamine; a synthetic CB1 analog). The TRP is overexpressed in several cancers such as melanoma, pancreatic cancer, prostate cancer and others, where it promotes survival of the cancer cells by making them resistant to ROS (redox adaptation) (84). The TRP antagonists increase sensitivity of cancer cells to chemotherapy-induced ROS production (94). By desensitizing TRPA-1, cannabinoids may increase sensitivity of cancer cells to ROS produced by chemotherapy and irradiation.

#### 5.5.7 VDAC-1

VDAC-1 is expressed on plasma membrane as well as on the outer mitochondrial membrane (95). By facilitating transfer of ions and metabolites across outer mitochondrial membrane, it regulates mitochondrial function, cell metabolism and apoptosis. In its open and high conductance state, VDAC-1 supports oxidative phosphorylation (OXPHOS), and in a closed low conductance state, it promotes aerobic glycolysis. In cancer cells, binding of hexokinase-II (HK-II) to VDAC-1 keeps it in the open state ensuring free supply of ATP for HK-II. CBD binds and displaces HK-II, inhibits OXPHOS, increases aerobic glycolysis and induces autophagy (96). CBD causes mitochondrial damage, fragmentation and swelling resulting in increased ROS production and apoptosis. CBG has similar but slightly different effects on cancer cell metabolism. *In vivo*, Epidiolex (CBD oil) and CBG in 1:1 ratio inhibit tumor growth of prostate cancer, neovascularization and Ki67+ cells (96). They disrupt mitochondrial membrane potential leading to the induction of apoptosis via mitochondrial or intrinsic pathway of apoptosis. Through this channel, CBD increase fission, decrease fusion and cause swelling of mitochondria (97).

### 5.6 Peroxisome proliferator-activated receptors

These are nuclear receptors, which upon binding with a ligand, heterodimerize with retinoid X receptor, and bind with PPAR-responsive DNA sequences and induce transcription of specific genes. They exist in three isoforms, α, β/δ and γ, and are involved in the regulation of lipid metabolism, energy homeostasis, adipogenesis, cell differentiation and inflammation. Cannabinoids act as ligands for α and γ isoforms, and upregulate their transcriptional activities; few studies have been performed with the β/δ isoform. Cannabinoids permeate plasma membrane and are chaperoned by intracellular fatty acid binding proteins (FABP)-3, 5 and 7. The FABP deliver the cannabinoids to PPARs. Through PPAR-γ, cannabinoids (CBD, THC, CBG and Ab-CBD) exert anti-tumor effects, promote neurogenesis and reduce neurodegeneration (98, 99). However, PPAR-γ is amplified in prostate cancer, and cannabinoids may promote the cancer progression through promoting mitochondrial biogenesis and fatty acid synthesis (100). Synthetic cannabinoids such as WIN-55,212 and HU210 exert nociceptive effects through PPAR-α and CB1 (101). As PPAR-γ agonists, cannabinoids, such as CBD and CBG, have promise as anti-diabetic drugs (102).

### 5.7 Serotonin receptors

They become dysregulated in several cancers such as prostate, breast, and glioma. Serotonin is a biogenic monoamine that acts as a local mediator in the gut, as a neurotransmitter in the brain and as a vasoactive agent in the vascular system. Serotonin receptors play a role in cancer cell proliferation, migration, metastasis and angiogenesis. Serotonin exerts its growth stimulating effects mainly via two receptors, 5-HT1_A_ and 5-HT2_A_ (103). Cannabinoids also exert their analgesic, antiemetic, anti-epileptic and hallucinogenic effects through 5-HT receptors (104, 105). CBD was recently shown to antagonize Lysergic acid diethylamide (LSD)-mediated Gαq activation and reduce LSD-mediated hallucigenic and psychotic effects (106). Despite the fact that THC acts as agonists and CBD acts as an inverse agonist for serotonin receptors, potential effect of the cannabinoids on cancer progression through serotonin receptors remain unexplored.

### 5.8 Glycine receptors

They are Cys-loop anion selective ligand-gated inhibitory ion channels. Other than their well-established role as neurotransmitters in the brain, GlyR also play a role in tumorigenesis in glioma and other brain tumors through their nuclear localization signals located in cytosolic loops (107). Knock-down of the GlyR results in reduced self-renewal capacity of the cancer cells (108). Cannabinoids such as THC potentiate these receptors, and cannabinoid-induced analgesia is absent in GlyR-KO mice but not in CB1 and CB2 lacking mice. They target GlyRα3 to reduce inflammatory and neuropathic pain (107). The receptors also occur in macrophages and their activation promotes M1 polarization. Acting through these receptors, cannabinoids could reduce cancer-associated pain, and regulate cancer progression.

### 5.9 N-methyl D-aspartate receptors

It is an ionotropic receptor for glutamate which is a potent excitatory neurotransmitter in the brain (109). The receptor is a heterotetramer comprising two R1 and either one of the four R2 (a-d) and two R3 (a, b) subunits. Glutamate along with co-agonists (glycine and D-serine) activates the receptor permeating Ca++, and to a lesser extent Na+ and K+. The receptors are located in the neuronal postsynaptic region of the glutamatergic synapses in the CNS as well in glial cells. The receptors also shift their locations from synaptic to extra-synaptic regions. Within the synapse, NMDAR activation performs important physiological functions including synaptic plasticity (important for learning and memory formation), behavioral learning and brain development. Persistent hypo or hyper functionality of NMDAR may lead to pathological conditions including such as depression, schizophrenia and autism spectrum disorder (ASD), etc. Extra-synaptic activation of the receptor leads to excitotoxicity and neuronal cell death and may lead to neurodegenerative diseases such as Alzheimer disease (AD), epilepsy and stroke. Within the CNS, NMDAR exist as complexes with CB1 and CB2 whose activation attenuates NMDAR’s excitatory signaling (110, 111). As stated above, CB2 is also expressed in certain neural cells in the brain. Interestingly, NMDAR activation leads to production of endocannabinoids in the brain that in turn inhibit NMDAR-mediated excitatory transmission. Endocannabinoids are one of the main mechanisms controlling over-activation of NMDAR. They also reduce pre-synaptic release of glutamate. A hypo or hyper functioning of the NMDAR is associated with psychiatric disorders such as schizophrenia, depression, neuropathic pain, mood disorders and autism, etc. Individuals who have genetically hypo-functional NMDAR, Cannabis smoking may exaggerate the hypo-functionality and trigger schizophrenia. Outside the CNS, NMDAR are also expressed in non-neuronal cells such as epithelial cells and immune cells, etc. In these cells, NMDAR are involved in cell-cell competition, in which metabolically better-fit cells survive and less-fit ones are eliminated (112).

Interestingly, NMDAR are also expressed in a wide variety of cancers including glioblastoma, prostate, breast, lung, gastro-intestinal tract (GIT), thyroid and pancreatic cancers (113). Mutated NMDAR subunits (especially R2) are found in several of these cancers. Cancers are well-documented to undergo metabolic reprograming (enhanced aerobic glycolysis; Warburg effect) and consume glutamine abundantly (glutamine addiction). They release glutamate (a metabolic product of glutaminolysis) that acts as a growth factor for cancer cells by acting through NMDAR. Interestingly, glutamate secreting cancer cells may also form glutamatergic synapses with neurons upon metastasis to brain. NMDAR antagonists such as memantine and MK801/dizocilpine have been widely used to control glutamate-induced cancer cell proliferation as well as metastasis in several cancer types. However, they are accompanied by severe side effects including in-coordinated mobility, catatonia, nightmares, hallucinations, social withdrawal and memory deficits. Phytocannabinoids may serve as better and safer choices to attenuate glutamate-mediated neurotoxicity and cancer progression. For example CBD normalizes the release of glutamate, cytokines and the induction of iNOS and COX-2 in cancer cells (114). It has also shown beneficial effects in epilepsy, Parkinson, MS and in psychiatric comorbidities (115).

## 6 Hetero-dimerization of cannabinoid receptors with other GPR

GPR are well known for their propensity to form heteromers with other GPR. Being GPR, CB1 and CB2 also form heterodimers with other GPR including human HER-2, CXCR4, adenosine receptor (A2_A_) and dopamine receptor D2, etc. The heterodimers, in response to cannabinoids, result in altered signaling cascades. The hetero-dimerization affects important physiological and pathological processes. For example, CB1 forms heterodimers with adenosine receptor A_2A_; motor depressant effects of a CB1 agonist were completely blocked by A2_A_ antagonists (116). CB2 forms heterodimers with HER2 and CXCR4. The hetero-dimerization with HER2 stabilizes the growth factor receptor and indicates poor prognosis for HER2+ cancers such as breast cancer. The dissociation of the heterodimer with CB2 agonist, THC, was shown to cause degradation of HER-2 and the cancer cell death (66). On the other hand, CB2 hetero-dimerization with CXCR4 affects migration of CXCR4+ hematopoietic cells. CB2 ligands inhibit migration, invasion and metastasis of CXCR4+ cancer cells (67, 117). CB1/D2 heteromers, when activated by CB1 agonists, induce signaling via Gαs, rather than Gαi protein, and activate adenylate cyclase and production of cAMP (118).

Overall, cannabinoids induce pain relieving, orexigenic, analgesic, anti-emetic, anxiolytic and anti-inflammatory effects through many of their canonical and non-canonical receptors. Many of these receptors also induce anti-tumor effects through their pro-apoptotic and metastasis inhibitory effects. However, cannabinoids also exert immunosuppressive effects. Furthermore, cancers have evolved strategies to evade anti-tumor effects of cannabinoids by upregulating pathways and/or accumulating variants of the receptors that promote their proliferation and spread. There are many studies that have investigated the effects of cannabinoids on different types of cancer. This review focuses at understanding the impact of cannabinoids on ICI immunotherapy in cancer patients. For the effects of cannabinoids on cancer, interested readers are referred to recent reviews (119–121).

## 7 Cannabinoids and ICI immunotherapy

ICIs are only effective in a subset of cancer types and patients and are often accompanied by ir-AE, which leads to suboptimal cancer therapies because of dose reductions or switches to traditional but less effective oncological treatments. There are preclinical and clinical studies which have shown the effect of cannabinoids on irAE.

### 7.1 Pre-clinical studies

In murine models, Xiong et al (122) reported that THC as well as AEA reduced efficacy of anti-PD1 therapy and impaired functional activities of the cancer specific T cells. In a FLAG-tagged CB2 knocked-in mouse, the authors showed that upon treatment with THC, CB2 bound with JAK-1 and inhibited STAT signaling in T cells. CB2KO in mice augmented anti-tumor immune responses. The authors recommended avoiding cannabinoids with ICIs. Both Cannabis-derived and endogenous cannabinoids inhibited T cell mediated immune responses. Compared with the vehicle (DMSO), THC increased tumor growth in MC38 and B-16 tumor models in mice (C57/Bl6). Anti-PD-1 decreased the growth and caused infiltration of CD8+ T cells in the TME; THC decreased the infiltration and secretion of IFN-γ from tumor-infiltrated T cells in *in vitro* assays. The effect of anti-PD1 in this model was mediated independently from macrophages and B cells. In *in vitro* studies, THC inhibited CD8+ T cell proliferation and production of IFN-γ and TNF-α. The authors measured AEA levels by ELISA in 170 lung cancer patients; the patients with high levels of AEA showed worst OS compared with low levels of AEA. Furthermore, high levels of CB2 by IHC also correlated with worse prognosis. Taken together, the authors concluded that the cannabinoids attenuated T cell-mediated antitumor immunity through CB2.

Experiments conducted in CB1KO and CB2KO mice by Sarsembayeva et al. (123) showed in a syngeneic mouse model of NSCLC that tumor growth was retarded in CB2 KO but not in CB1 KO mice. Anti-PD-1 was more effective in reducing tumor progression and causing infiltration of T and NK cells in the TME in CB2 KO mice. The authors found that leukocytes in the TME of melanoma, NSCLC and clear cell renal carcinoma patients showed high expression of CB2. Endogenous cannabinoids, acting through CB2, reduced cytotoxicity of NK cells and CTL against the cancer.

More recently, the effects of medicinal Cannabis concomitantly with ICIs were reported in metastatic NSCLC (124); the cancer that accounts for close to 80% of the lung cancer cases. The authors investigated the effects of a Cannabis preparation containing 68% THC, 2% CBG, 1% CBD and 1% CBN with and without anti-PDL-1 antibody (Pembrolizumab) in murine colorectal carcinoma cell line (CT26)-bearing female Balb/C mice. The preparation was injected intraperitoneal beginning at the time of the tumor implantation. The combination therapy significantly (p<0.05) increased OS in mice compared with anti-PD1 and cannabinoid monotherapies. The Cannabis treatment reduced anti-PD1-mediated infiltration of CD4+ T cells in the tumors by 21%; the difference, however, did not reach significance.

### 7.2 Clinical studies

In a retrospective and observational study on 140 patients suffering from melanoma, NSCLC or RCC, Taha et al. (125) found that in the 51 patients on Nivolumab plus cannabis, progression free survival (PFS) and overall survival (OS) were not affected, whereas the response rate (RR) was significantly reduced (**Table 3**). Nevertheless, a better RR was found in patients consuming cannabis products with a higher THC content. Humanized Nivolumab prevents PD1-PDL1 interaction and causes tumor cell death by invigorating effector T cells and by down-regulating Tregs.

**Table 3:** Studies investigating the impact of cannabis in conjunction with ICIs in cancer patients.

Authors (Ref #)	Study Design	Participants/Model	Cannabinoids (Type and Mode of Consumption)	Main Results	Conclusions
Taha et al. 2019 (125)	Retrospective observational	140 patients with advanced melanoma, NSCLC and RCC treated with Nivolumab; 51 cannabis users (78.4% males); 89 non-cannabis users	Cannabis products containing varying percentages of THC and CDB; inhaled/smoked or took orally	THC and CBD did not affect RR; better RR in patients with higher THC content product; no effect on PFS; smoking status and brain metastasis factors in PFS; Cannabis affected OS in univariate but not in multivariate analysis	Cannabis use reduces RR to ICI therapy but does not significantly affect PFS or OS
Bar-Sela et al., 2020 (126)	Prospective observational	102 cancer patients (78% males) with metastatic malignancies (stage IV disease); NSCLC (50%), melanoma (37%) and others; 34 cannabis users and 68 non-cannabis users	71% patients consumed 20 grams/month, 10 consumed patients used 30-40 grams per month; consumption started 2-9 months prior to immunotherapy; products were oil with or without flower; took orally or by inhalation	Cannabis users showed disease progression; median TTP for cannabis users was 3.4 months versus 13.1 months for non-users; significantly better TTP and OS in non-users; ir-AE were significantly reduced in cannabis users; users had 16% more cases of lower lymphocyte counts compared with baseline; ICI altered blood endocannabinoid levels but changes between users and non-users were not different	In metastatic malignancies, cannabinoid use caused a significant reduction in TTP and OS but reduced ir-AE
Biedny et al., 2020 (127)	Retrospective	104 patients with advanced-stage cancers (41.3% lung adenocarcinoma, 20.3% lung squamous cell carcinoma, 11.5% HNSCC, 26.9% other tumor types) who received 10.2 months of immune checkpoint inhibitors (Nivolumab or Pembrolizumab during 2014-2018), 48.1% males, 26.9% cannabis users	23 patients were prescribed Dronabinol orally, 5 patients consumed recreational cannabis; smoked or orally	Non-cannabis users had a significantly longer OS when compared with cannabis users (40 months vs. 16 months, p=0.0004)	When used in conjunction with ICI immunotherapy, cannabis consumption decreases OS
Xiong et al., 2022 (122)	Retrospective, and experimental using CB2 knocked-in mice	Determined serum levels of AEA, expression of CB2 and correlated with progression of lung cancer in patients; effect of THC and AEA in mice on CD8+ T cells was investigated in B16 melanoma and MC38 colon adenocarcinoma pretreated with anti-PD-1 and then with cannabinoids; effect was also investigated on adoptive transfer of cancer-specific CD8+ T cells in a mouse model grafted with OVA-expressing cancer grafts; the researchers generated a conditional knocked in mouse expressing EGFP- and a FLAG-tagged CB2 in T cells; measured effects of THC and AEA on T cells in lymph nodes and spleen and on tumor growth	THC and AEA injected in mice	Higher serum AEA levels correlated with worse OS in cancer patients; in mice THC and AEA significantly inhibited CD8+ T cell proliferation, TNF-α and IFN-γ expression, accelerated tumor growth and diminished effects of anti-PD 1. Ablation of CB2 promoted T cell proliferation without any effect on their negative selection; Tumors showed slower growth and better survival in CB2 KO mice; TILs were more activated with less exhaustion markers	Cannabis and its constituent (THC) and AEA increase tumor progression and reduce anti-tumor effects of CD8+ T cells ICI immunotherapy. Simultaneous CB2 antagonism reverses pro-tumor effects of cannabinoids.
Sarsembayeva et al., 2023 (123)	Experimental study in mouse model and human patients	Lymphocytes from TME of NSCLC mouse model and human biopsies; experimental studies in CB1 KO and CB2 KO mice with anti-PD-1 antibodies	Administered anti-PD-1 antibodies in CB1-KO and CB2-KO mice with syngeneic NSCLC	Leukocytes from TME expressed higher levels of CB2 than CB1; tumor progression retarded in CB2KO mice but not in CB1 KO mice; anti-PD-1 attenuated tumor growth in CB2KO mice, and caused more infiltration of T and NK cells	CB2 exerts pro-tumor effects by suppressing cytotoxicity of CD8+ T cells and NK cells. Simultaneous antagonism of CB2 enhances anti-tumor effects of anti-PD-1 therapy
Waissengrin et al., 2023 (124)	Retrospective observational study; experimental study using C26 tumor-bearing mouse model	201 patients with metastatic NSCLC treated with pembrolizumab as first line treatment; 102 patients (50.7%) cannabis users, users were younger than non-users (median 68 vs 74 years); 60.8% females with higher rates of brain and liver metastasis; mice were treated with anti-PD-1 with and without a cannabis preparation containing 68% THC	49% patients consumed cannabis orally (oil extracts); 51% inhaled cannabis; median monthly dose 30g; 36% patients used the oil with 10% THC & 2% CBD	TTP was similar for cannabis-naïve and cannabis using patients (6.1 vs 5.6 months; OS was higher in cannabis naïve group (54.9 vs 23.6 months) but did not reach statistical significance; cannabis use was not an independent predictor for mortality in multivariate analysis. OS of mice receiving vehicle, THC, anti-PD1 antibody or their combination was 21, 24, 31, and 54 days, respectively (combination vs control <0.05)	No deleterious effect of cannabis consumption on activity of pembrolizumab therapy in advanced NSCLC patients; THC did not reduce efficacy of anti-PD1 therapy in murine model

ALL, Acute Lymphoblastic Leukemia; EC, Endocannabinoids; HNSCC, Head and neck squamous cell carcinoma; HPV, Human Papillomavirus; NSCLC, Non-small cell lung cancer; OS, Overall survival; OVA, Ovalbumin; PFS, Progression free survival; RCC, Renal cell carcinoma; RR, Response rate; TILs, Tumor-infiltrating T cells; TTP, Time to tumor progression; VEC, Vascular endothelial cell.

Bar-Sela et al. (126) supported these findings in a prospective and observational study involving 102 advanced cancer patients (RCC, melanoma, NSCLC) receiving ICIs; 34 of whom consumed cannabis. They found that in metastatic malignancies, cannabinoid use caused a significant reduction in time to tumour progression (TTP) and OS, but significantly improved ir-AE such as skin toxicity, thyroid disorders, colitis, renal insufficiency, arthritis and hepatitis. The authors also measured the concentrations of circulating endocannabinoids and endocannabinoid-like lipids using liquid chromatography/mass spectrometry before and after immunotherapy in both groups. Interestingly, the cannabis users had reduced levels of endocannabinoids in their circulation which correlated with OS. The lower levels of endocannabinoids in the circulation of cannabis users were not expected as THC and CBD are known to enhance these levels by competitive binding with FABP and preventing degradation of endocannabinoids (46).

Biedny et al. (127) also found in a retrospective study involving 104 patients with advanced-stage malignancy (41.3% lung adenocarcinoma, 20.3% lung squamous cell carcinoma, 11.5% head and neck squamous cell carcinoma (HNSCC), and 26.9% other tumor types) treated with Nivolumab or Pembrolizumab for an average of 10.2 months, that cannabis users (who were more frequently smokers), had significantly shorter OS as compared with cannabis non-users.

On the other hand, Waissengrin et al. (124) found that in 102 metastatic NSCLC patients, THC did not reduce efficacy of anti-PD1 immunotherapy. Although OS was higher in THC-naïve patients, it did not reach significance. In multivariate analysis, THC did not reduce efficacy of anti-PD1 monotherapy. It is noteworthy that THC users were younger, predominantly females and had more brain metastases, a site where THC is more effective. The authors reported no significant difference in the expression of PDL-1 in tumors between THC and non-THC users; all were positive for PDL-1. Time to tumor progression (TTP) was shorter in the cannabis users, however, the OS was not significantly shorter in the latter as compared to cannabis nonusers. It was concluded that cannabis does not have detrimental effects on ICI therapy.

In summary, the clinical studies available to date suggest that cannabinoids are able to minimize ir-AEs, however, the reductions in OS, TTP and RR were found either not uniform or not significant across the available studies. Nevertheless, important methodological limitations make the interpretation of the above results challenging. Most of these studies were retrospective, various cannabinoid products were used with unclear dosages and most of the patients considered had already advanced-metastatic diseases. Randomized placebo-controlled trials with adequate power and standardized preparations of cannabinoids are needed.

## 8 Cannabinoids’ effects on pharmacokinetics and pharmacodynamics of ICIs

ICIs are mainly administered intravenously and are distributed rapidly into various body tissues and fluids. However, their access to tumor microenvironment depends upon the latter’s composition. Being monoclonal antibodies, ICIs are catabolized by proteolytic enzymes in the body with half-lives ranging from several days to a few weeks (131). As ICIs are not metabolized in the body by CYP450 enzymes, cannabinoids are not likely to affect their pharmacokinetics. Thus, xenobiotics like cannabinoids have little capacity to affect pharmacokinetics (absorption, distribution, metabolism and excretion) of ICIs. Cannabinoids, however, could potentially modulate pharmacodynamics of ICIs. In CRC, CBD was shown to rewire TME, decreases alternate activation of macrophages and increase expression of IFN-γ and IFN-α. It also enhanced efficacy of anti-PD-1 ICIs through increased expression of PDL-1 (128). Furthermore, CBD was reported to stimulate the expression of PDL-1 through activating cGAS-STING pathway in triple negative breast cancer cells. It also enhanced efficacy of Atezolizumab, an anti-PDL-1 ICI (129). In contrast, CBD and/or THC were shown to reduce PDL-1 expression by pancreatic cancer and pancreatic stellate cells (130). These studies suggest cancer-type specific effects of cannabinoids on the expression of immune checkpoints. Clearly, further research is needed on this topic.

## 9 Factors that modulate cannabinoids’ effects on cancer and ICI immunotherapy

Despite anti-cancer effects of cannabinoids demonstrated in several *in vitro* studies and animal models, such beneficial effects have not been consistently observed in human patients (132). The discordance may be attributed to variations in cancer types, treatment regimens, patient’s genetics, and microbiota. The same variables may lead to divergent results in the studies examining the impact of cannabinoids in conjunction with ICIs. The variables are discussed below.

### 9.1 Repertoire of cannabinoid receptors

The anti-cancer effects of cannabinoids depend upon the repertoire of both classical and non-classical cannabinoid receptors as well as the integrity of their signaling pathways expressed by the cancer cells. The expression of the receptors depends upon the type of cancer and may change with the stage of cancer development (92, 133). Furthermore, as discussed above, the GPCR such as CB1 and CB2 form heterodimers with other GPCR such as HER-2, CXCR4 and others that have implications for cancer progression (66, 67). Importantly, cancers may also select for mutations in the in the cannabinoid receptor genes as well as in the genes involved in the cannabinoid-induced signaling (134–137). There is need for investigating cannabinoid receptor variants associated with different types of cancers.

### 9.2 Microbiome

It is well recognized that gut microbiome impacts most physiological and pathological processes including carcinogenesis in our body. Certain microbiota and their metabolites are known to promote the development and progression of cancer (138, 139). Changes in the gut microbiota composition as well as diversity may result from metabolic changes and adaptations imposed by the cancer. In general, the relative abundance of pro-inflammatory and carcinogenic *Prevotella, Parasuterella, Hungatella, Sneathia* and *Fusobacterium* species increases in the gut of cancer patients whereas that of short chain fatty acids (SCFA)-producing anti-inflammatory bacteria such as *Anaerostipes caccae* decreases (140). Recent studies have demonstrated that gut, oral, and skin microbiota, as well as tumor-infiltrating microbiota are associated with patients’ responses to ICI therapy (141). Fecal transplantation from ICI responders into anti-PD-1 refractory melanoma improved responses in 30-40% of the patients (142). In this context, *Akkermansia muciniphila* and the bacteria belonging to the Actinobacteria and Fermicutes phyla are associated with higher responsiveness to ICIs. On the other hand, a relative abundance of *Bacteroides clarus* was found in non-responders (141). Gut microbiota is very dynamic and many factors, in addition to diet, may affect its composition. The potential role of cannabinoids in modulating the gut and tumor microbiota is important. Many commensals in the gut produce N-acylamides, small endocannabinoid mimetics that specifically bind to certain GPCR and regulate gut physiology (143). For example, *Akkermansia muciniphila*; a bacterial species known for its many host-beneficial effects, produces 2-AG, 1-PG and 2-PG (144, 145). Furthermore, they also produce β-glucuronidase, and convert inactive glucuronidated metabolites of cannabinoids back into active metabolites that are absorbed into the body through intestines. Many gut microbiota produce enzymes that can metabolize cannabinoids such as THC and CBD into both active and inactive metabolites (145). They are also implicated in the production of secondary bile salts, which form complexes with cannabinoids and affect their bioavailability, metabolism and efficacy. Thus, gut microbiota exquisitely regulate endocannabinoid system as well as phytocannabinoids in the body. The cannabinoids, in turn, are known to affect gut microbial composition (146). In a recent study, THC was shown to reduce gut dysbiosis and neuroinflammation in SIV-infected *Rhesus macaques (* 147). It concurrently increased the relative abundance of Fermicutes, Clostridia, Lactobacilli and Bifidobacteria in the colon of THC-treated animals. The increase was noteworthy in bacterial species producing SCFA and indole-3-propionate. In contrast, a decreased relative abundance of a dysbiotic species *Escherichia fecalis* was observed. THC also increased plasma levels of endocannabinoids (147). In humans, however, the cannabis consumption was associated with increased relative abundance of Bacteroides species, which might cause increased inflammation and metabolic disorders (148). Therefore, simultaneous consumption of pre- and pro-biotics may be helpful. The mechanisms through which exogenous cannabinoid consumption affects gut microbiome in cancer patients undergoing chemotherapy and immunotherapy remains largely un-explored. Future research efforts should be directed at understanding biological pathways through which ICIs and cannabinoids affect gut microbiota and vice versa.

### 9.3 Cannabinoid preparations

Effects of consumed cannabinoids may depend upon type of preparation and its route of administration (149). Crude cannabis preparations contain numerous compounds (cannabinoids, terpenoids, flavonoids, phenols and other phytochemicals, many of which may act in synergism ‘entourage effect’. It may be difficult to interpret their results. Most isolated or purified cannabinoids such as THC, CBD or CBG are administered orally alone or combined in different ratios. They have low bioavailability, however, their results could be consistent and interpretable. Efforts are underway to develop nano-formulations of cannabinoids for more reliable delivery systems and for better pharmacokinetics; it may become possible to use them peri-tumorally (150).

### 9.4 Polymorphism in cannabinoid-metabolizing enzymes

It is noteworthy that CYP450 genes are highly polymorphic and show haplotypic variation. Humans could be divided into four phenotypes: ultra-rapid metabolizers (UM), who inherit more than two copies of active CYP genes, extensive metabolizers (EM) or normal metabolizers, who inherit two active CYP genes, intermediate metabolizers (IM) who inherit one functional and one defective gene, and poor-metabolizers (PM) in whom both copies of CYP450 genes are defective. About 15-20% of humans carry 2C9*3 allele, which metabolizes THC poorly. The individuals with 2C9*3/*3 genotype (PM) accumulate 200-300 times more THC in their blood and are more likely to experience an increased duration and intensity of THC intoxication, especially when taking THC orally. With respect to CYP2C19, about 25% of the individuals carry the gene variants that metabolize CBD slowly than normal individuals. Conversely, 25% of individuals are fast metabolizers of CBD. Rare UM individuals can metabolize and eliminate cannabinoids very rapidly and usual doses may not provide desirable clinical results in them (151). On the other hand, in PM, cannabinoids may cause toxicity, and therefore dose adjustments would be required.

### 9.5 Drug-drug interactions

By acting as substrates for CYP450 enzymes, cannabinoids may inhibit them through competitive or non-competitive ways. Furthermore, they could induce certain of these enzymes through their transcriptional activation. By inhibiting or inducing these enzymes, cannabinoids affect the metabolism of other drugs. Major cannabinoids such as THC and its metabolites (11-OH-THC and 11-nor-9-COOH-THC-Gluc), CBD and CBN competitively inhibit CYP2B6, CYP2C9, CYP2D6 CYP2C19, CYP2A1, CYP1A2 and CYP3A4/5 to variable extents (51, 52). As CYP450 enzymes also play an essential role in the production of steroids, cholesterol and prostanoids, their inhibition may result in toxicities. About 80% of conventionally used drugs are metabolized by 3A4/5, 2C19, 2C9 and 2D6. They include anti-cancer drugs such as Tamoxifen, Doxorubicin, Cyclophosphamide and Ifosamide, as well as many non-cancer drugs such as Acetaminophen. Concurrent consumption of cannabinoids with these medications may result in drug-drug interactions with an impact on their effectiveness. For example, CBD is known to inhibit CYP2D6 and CYP3A4, the enzymes that metabolize Tamoxifen, a prodrug used as an endocrine therapy in breast cancer, into its active metabolite Endoxifen (152). Concurrent usage/administration of cannabinoids with Tamoxifen may lead to suboptimal concentrations of Endoxifen in breast cancer patients and treatment failure (153). CBD oil is often used by the breast cancer patients for relief from Tamoxifen-induced hot flashes, arthralgia, insomnia, and mood alterations. On the other hand, CBD and Tamoxifen were shown to exert synergistic effects in targeting mitochondria and killing T-ALL cells; T-ALL is a less common but highly aggressive hematological malignancy (154). CBD was also demonstrated to increase anti-cancer cytotoxicity of several drugs such as Cisplatin, 5-Fluorouracil, Paclitaxel and Doxorubicin in mouse models of HNSCC (155). Finally, cannabinoids could also induce certain CYP450 enzymes. CBD acts as an inducer of 1A2, 2B6 and 3A4 whereas THC induces 1A1 and 2C9 (51, 156). Drugs that are metabolized by these enzymes are more rapidly metabolized in the body and require dose adjustments.

### 9.6 Impact on anti-cancer immune response

Cannabinoids are known to exert immunosuppressive effects. Both CB1 and CB2 agonists attenuate cell mediated immunity (157). They reduce the expression of activation markers and co-stimulatory molecules in immune cells, impair T cell proliferation and cytokine production and diminish CTL-mediated cytotoxicity (157). CB2 selective cannabinoids also promote development of Tregs and production of IL-10 (158). It was demonstrated that upon binding with agonist cannabinoids, such as THC, CB1 bind JAK-1 and inhibits downstream STAT-mediated signaling (122). THC also inhibits CD3/CD28-mediated CD8+ T cell proliferation *in vitro* as determined by the carboxylfluorescein diacetate succinimidyl ester (CFSE) dilution assay, inhibits their effector functions i.e., cytotoxicity and production of IFN-γ and TNF-α (159). Studies in mice have shown that T cell specific CB2 deficiency promotes T cell development. Mice lacking T cell-specific CB2, showed increased numbers of CD4+ and CD8+ T cells in the thymus, whereas double positive T cells were slightly increased. Thus CB2 ablation promoted T cell development. THC and AEA did not inhibit proliferation of T cells from CB2-deficient mice but did so for T cells from wild type mice. T cells from CB2 KO mice showed enhanced production of IFN-γ and decreased expression of CD39, PD-1 and LAG-3; the deficiency also enhanced expansion and function of CD8+ T cells in the tumor microenvironment (160). Future studies should take into consideration cannabinoids’ effects on ICI immunotherapy and anti-cancer immunity. Given an evident adverse effect of CB2 selective cannabinoids on anti-cancer immune responses, simultaneous inhibition of this receptor might be desirable.

### 9.7 HLA repertoire

The response of a cancer patient to ICI immunotherapy is, at least in part, dependent upon his/her repertoire of HLA-class I and -class II alleles as well as their levels of expression. For example, HLA-A*03 and HLA-B66 super types are associated with lower responses whereas HLA-B44 super types are associated with better responses to ICI immunotherapy (161). The associations depend upon the ability of the HLA molecules to present cancer-associated neo-antigens. Downregulation of HLA is a common immune evasion strategy used by cancer cells. Interestingly, phytocannabinoids (THC, CBD and CBG), but not endocannabinoids, were recently shown to upregulate the expression of HLA-class I molecules on the surface of several metastatic cancer cells; the upregulation was not mediated through CB1 or CB2 (162).

## 10 Conclusions

Cannabinoids may relieve pain, nausea, anorexia and anxiety, and improve quality of life in cancer patients. ICI immunotherapy, although very successful in a subset of cancer patients, is accompanied by moderate to severe ir-AE that necessitate discontinuation of the immunotherapy in many patients; the ir-AE may persist even after discontinuation of the therapy (1). Because of the anti-inflammatory and immunosuppressive effects of cannabinoids, researchers have contemplated their use in combination with ICI immunotherapy. Results from a few studies conducted so far strongly suggest that the use of medicinal cannabis in cancer patients attenuates many of the ir-AE associated with the use of ICIs and increase its tolerability. However, no significant beneficial effects on overall survival, progression free survival or cancer relapses were observed; rather some of the studies noted adverse effects of concurrent administration of cannabinoids with the immunotherapy on clinical outcomes (163). Because of cannabinoids’ well documented immunosuppressive effects mediated through CB2 (122), we propose considering these molecules as an inhibitory immune checkpoints. A simultaneous neutralization of this checkpoint may lead to better clinical outcomes in cancer patients receiving ICIs concurrent with cannabinoid treatment. In this regard, cannabinoids such as CBD and CBG, with little agonism for CB2, may be better therapeutic choices. They also lack psychotoxic effects of THC. Additional strategies e.g., the use of MAGL inhibitors that decrease lipogenesis and formation of lipid bilayers in cancer cells (164) may also be explored. They increase concentrations of 2-AG and their congeners in the body. However, they liberate AA which is metabolized by COX into pro-inflammatory prostaglandins (PG). Furthermore COX also metabolize 2-AG to produce PG-glycerols. These COX-generated lipid mediators counter some of the endocannabinoids/phytocannabinoids’ beneficial effects on cancer progression and hypo-algesia. A simultaneous use of cannabinoids and COX-inhibitors may yield better results and should be explored. Future studies should take into consideration CYP450 genotypes and haplotypes and cannabinoid-drug interactions for personalized cannabis-based therapies in cancer patients receiving cannabinoids along with ICIs. This may lead to rational knowledge-based regimens tailored to individual cancer patients. Despite several studies demonstrating anti-cancer effects of cannabinoids primarily in pre-clinical models, these effects were not confirmed in cancer patients. It is very difficult to reconcile results from different studies due to heterogeneous nature of the cancers studied, unstandardized cannabinoid preparations, populations of patients with already advanced disease stages, different genetic makeup of cancer patients with respect to their HLA genotypes and CYP450 genotypes and haplotypes, patients’ unique gut microbiota as well as different genetic and somatic variations occurring in the cannabinoid receptors and their signaling pathways in the cancer cells. Unfortunately, many of the studies that investigated the impact of cannabinoids on cancer progression were carried out in the absence of anti-cancer immune responses. Finally, purified and standardized cannabinoids such as CBD and CBG that lack psychoactive effects should be investigated should be investigated in cancer patients receiving ICI immunotherapy.

## References

[1] Groupe de Recherche en Reanimation Respiratoire du patient d’Onco-Hématologie (Grrr-OH) Lemiale V Meert A-P Vincent F Darmon M Bauer PR. Severe toxicity from checkpoint protein inhibitors: what intensive care physicians need to know? Ann Intensive Care. (2019) 9:25. doi: 10.1186/s13613-019-0487-x 30707321 PMC6358632

[2] Alturki NA. Review of the immune checkpoint inhibitors in the context of cancer treatment. JCM. (2023) 12:4301. doi: 10.3390/jcm12134301 37445336 PMC10342855

[3] Guo Z Zhang R Yang A-G Zheng G. Diversity of immune checkpoints in cancer immunotherapy. Front Immunol. (2023) 14:1121285. doi: 10.3389/fimmu.2023.1121285 36960057 PMC10027905

[4] Ozbay Kurt FG Lasser S Arkhypov I Utikal J Umansky V. Enhancing immunotherapy response in melanoma: myeloid-derived suppressor cells as a therapeutic target. J Clin Invest. (2023) 133:e170762. doi: 10.1172/JCI170762 37395271 PMC10313369

[5] Wei SC Duffy CR Allison JP. Fundamental mechanisms of immune checkpoint blockade therapy. Cancer Discovery. (2018) 8:1069–86. doi: 10.1158/2159-8290.CD-18-0367 30115704

[6] Mansh M. Ipilimumab and cancer immunotherapy: A new hope for advanced stage melanoma. Yale J Biol Med. (2011) 84:381–9. PMC3238313 22180676

[7] Stirling ER Bronson SM Mackert JD Cook KL Triozzi PL Soto-Pantoja DR. Metabolic implications of immune checkpoint proteins in cancer. Cells. (2022) 11:179. doi: 10.3390/cells11010179 35011741 PMC8750774

[8] Van Der Kraak L Goel G Ramanan K Kaltenmeier C Zhang L Normolle DP. 5-fluorouracil upregulates cell surface B7-H1 (PD-L1) expression in gastrointestinal cancers. J immunotherapy Cancer. (2016) 4:65. doi: 10.1186/s40425-016-0163-8 PMC5067917 27777774

[9] Pu Y Ji Q. Tumor-associated macrophages regulate PD-1/PD-L1 immunosuppression. Front Immunol. (2022) 13:874589. doi: 10.3389/fimmu.2022.874589 35592338 PMC9110638

[10] Wang Q Xie B Liu S Shi Y Tao Y Xiao D. What happens to the immune microenvironment after PD-1 inhibitor therapy? Front Immunol. (2021) 12:773168. doi: 10.3389/fimmu.2021.773168 35003090 PMC8733588

[11] Niu M Liu Y Yi M Jiao D Wu K. Biological characteristics and clinical significance of soluble PD-1/PD-L1 and exosomal PD-L1 in cancer. Front Immunol. (2022) 13:827921. doi: 10.3389/fimmu.2022.827921 35386715 PMC8977417

[12] Wang X Yang X Zhang C Wang Y Cheng T Duan L. Tumor cell-intrinsic PD-1 receptor is a tumor suppressor and mediates resistance to PD-1 blockade therapy. Proc Natl Acad Sci USA. (2020) 117:6640–50. doi: 10.1073/pnas.1921445117 PMC7104341 32161124

[13] Zhang H Dutta P Liu J Sabri N Song Y Li WX. Tumour cell-intrinsic CTLA 4 regulates PD -L1 expression in non-small cell lung cancer. J Cell Mol Medi. (2019) 23:535–42. doi: 10.1111/jcmm.13956 PMC6307812 30378264

[14] Kennedy PT Saulters EL Duckworth AD Lim YJ Woolley JF Slupsky JR. Soluble CTLA-4 attenuates T cell activation and modulates anti-tumor immunity. Mol Ther. (2024) 32:457–68. doi: 10.1016/j.ymthe.2023.11.028 PMC10861965 38053333

[15] Oh SY Kim S Keam B Kim TM Kim D-W Heo DS. Soluble PD-L1 is a predictive and prognostic biomarker in advanced cancer patients who receive immune checkpoint blockade treatment. Sci Rep. (2021) 11:19712. doi: 10.1038/s41598-021-99311-y 34611279 PMC8492653

[16] Cheng W Kang K Zhao A Wu Y. Dual blockade immunotherapy targeting PD-1/PD-L1 and CTLA-4 in lung cancer. J Hematol Oncol. (2024) 17:54. doi: 10.1186/s13045-024-01581-2 39068460 PMC11283714

[17] Lou H Cai H Huang X Li G Wang L Liu F. Cadonilimab combined with chemotherapy with or without bevacizumab as first-line treatment in recurrent or metastatic cervical cancer (COMPASSION-13): A phase 2 study. Clin Cancer Res. (2024) 30:1501–8. doi: 10.1158/1078-0432.CCR-23-3162 PMC11016896 38372727

[18] Pang X Huang Z Zhong T Zhang P Wang ZM Xia M. Cadonilimab, a tetravalent PD-1/CTLA-4 bispecific antibody with trans-binding and enhanced target binding avidity. mAbs. (2023) 15:2180794. doi: 10.1080/19420862.2023.2180794 36872527 PMC10012886

[19] Keenan TE Burke KP Van Allen EM. Genomic correlates of response to immune checkpoint blockade. Nat Med. (2019) 25:389–402. doi: 10.1038/s41591-019-0382-x 30842677 PMC6599710

[20] Das R Bar N Ferreira M Newman AM Zhang L Bailur JK. Early B cell changes predict autoimmunity following combination immune checkpoint blockade. J Clin Invest. (2018) 128:715–20. doi: 10.1172/JCI96798 PMC5785243 29309048

[21] Francisco LM Sage PT Sharpe AH. The PD-1 pathway in tolerance and autoimmunity. Immunol Rev. (2010) 236:219–42. doi: 10.1111/j.1600-065X.2010.00923.x PMC2919275 20636820

[22] Choi J Lee SY. Clinical characteristics and treatment of immune-related adverse events of immune checkpoint inhibitors. Immune Netw. (2020) 20:e9. doi: 10.4110/in.2020.20.e9 32158597 PMC7049586

[23] Wang DY Salem J-E Cohen JV Chandra S Menzer C Ye F. Fatal toxic effects associated with immune checkpoint inhibitors: A systematic review and meta-analysis. JAMA Oncol. (2018) 4:1721. doi: 10.1001/jamaoncol.2018.3923 30242316 PMC6440712

[24] Bessede A Marabelle A Guégan JP Danlos FX Cousin S Peyraud F. Impact of acetaminophen on the efficacy of immunotherapy in cancer patients. Ann Oncol. (2022) 33:909–15. doi: 10.1016/j.annonc.2022.05.010 35654248

[25] Johnson DB. Toxicities and outcomes: do steroids matter? Cancer. (2018) 124:3638–40. doi: 10.1002/cncr.31627 PMC6214744 29975416

[26] Li J Yang K Zhao L Bai C Sun Z. Impact of corticosteroids use on efficacy of immune checkpoint inhibitors in cancer patients: A meta-analysis. JCO. (2020) 38:e15234–4. doi: 10.1200/JCO.2020.38.15_suppl.e15234

[27] Radwan MM Chandra S Gul S ElSohly MA. Cannabinoids, phenolics, terpenes and alkaloids of cannabis. Molecules. (2021) 26:2774. doi: 10.3390/molecules26092774 34066753 PMC8125862

[28] Maldonado R Cabañero D Martín-García E. The endocannabinoid system in modulating fear, anxiety, and stress. Dialogues Clin Neurosci. (2020) 22:229–39. doi: 10.31887/DCNS.2020.22.3/rmaldonado PMC7605023 33162766

[29] Tahir MN Shahbazi F Rondeau-Gagné S Trant JF. The biosynthesis of the cannabinoids. J Cannabis Res. (2021) 3:7. doi: 10.1186/s42238-021-00062-4 33722296 PMC7962319

[30] Walsh KB McKinney AE Holmes AE. Minor cannabinoids: biosynthesis, molecular pharmacology and potential therapeutic uses. Front Pharmacol. (2021) 12:777804. doi: 10.3389/fphar.2021.777804 34916950 PMC8669157

[31] Geci M Scialdone M Tishler J. The dark side of cannabidiol: the unanticipated social and clinical implications of synthetic Δ^8^ -THC. Cannabis Cannabinoid Res. (2022) 8:270–82. doi: 10.1089/can.2022.0126 PMC10061328 36264171

[32] Graziano S Varì MR Pichini S Busardo FP Cassano T Di Trana A. Hexahydrocannabinol pharmacology, toxicology, and analysis: the first evidence for a recent new psychoactive substance. Curr Neuropharmacol. (2023) 21:2424–30. doi: 10.2174/1570159X21666230623104624 PMC10616920 37357519

[33] González-Mariscal I Pozo-Morales M Romero-Zerbo SY Espinosa-Jimenez V Escamilla-Sánchez A Sánchez-Salido L. Abnormal cannabidiol ameliorates inflammation preserving pancreatic beta cells in mouse models of experimental type 1 diabetes and beta cell damage. BioMed Pharmacother. (2022) 145:112361. doi: 10.1016/j.biopha.2021.112361 34872800

[34] Barcaccia G Palumbo F Scariolo F Vannozzi A Borin M Bona S. Potentials and challenges of genomics for breeding cannabis cultivars. Front Plant Sci. (2020) 11:573299. doi: 10.3389/fpls.2020.573299 33101342 PMC7546024

[35] Hazekamp A Fischedick JT. Cannabis - from cultivar to chemovar. Drug Testing Anal. (2012) 4:660–7. doi: 10.1002/dta.407 22362625

[36] de Brito Siqueira ALG Cremasco PVV Bahú JO Pioli da Silva A Melo de Andrade LR González PGA. Phytocannabinoids: pharmacological effects, biomedical applications, and worldwide prospection. J Tradit Complement Med. (2023) 13:575–87. doi: 10.1016/j.jtcme.2023.08.006 PMC10658372 38020546

[37] Brunt TM Bossong MG. The neuropharmacology of cannabinoid receptor ligands in central signaling pathways. Eur J Neurosci. (2022) 55:909–21. doi: 10.1111/ejn.14982 PMC9291836 32974975

[38] Peters EN MacNair L Mosesova I Christians U Sempio C Klawitter J. Pharmacokinetics of cannabichromene in a medical cannabis product also containing cannabidiol and Δ9-tetrahydrocannabinol: A pilot study. Eur J Clin Pharmacol. (2022) 78:259–65. doi: 10.1007/s00228-021-03232-8 PMC8748343 34664109

[39] Devane WA Dysarz FA Johnson MR Melvin LS Howlett AC. Determination and characterization of a cannabinoid receptor in rat brain. Mol Pharmacol. (1988) 34:605–13. doi: 10.1016/S0026-895X(25)09876-1 2848184

[40] Hillard CJ. Circulating endocannabinoids: from whence do they come and where are they going? Neuropsychopharmacology. (2018) 43:155–72. doi: 10.1038/npp.2017.130 PMC5719092 28653665

[41] Costiniuk CT Jenabian M-A. Cannabinoids and inflammation: implications for people living with HIV. AIDS. (2019) 33:2273–88. doi: 10.1097/QAD.0000000000002345 31764093

[42] Shamran H Singh NP Zumbrun EE Murphy A Taub DD Mishra MK. Fatty acid amide hydrolase (FAAH) blockade ameliorates experimental colitis by altering microRNA expression and suppressing inflammation. Brain Behav Immun. (2017) 59:10–20. doi: 10.1016/j.bbi.2016.06.008 27327245 PMC5154806

[43] Zanfirescu A Nitulescu G Mihai DP Nitulescu GM. Identifying FAAH inhibitors as new therapeutic options for the treatment of chronic pain through drug repurposing. Pharm (Basel). (2021) 15:38. doi: 10.3390/ph15010038 PMC8781999 35056095

[44] Gil-Ordóñez A Martín-Fontecha M Ortega-Gutiérrez S López-Rodríguez ML. Monoacylglycerol lipase (MAGL) as a promising therapeutic target. Biochem Pharmacol. (2018) 157:18–32. doi: 10.1016/j.bcp.2018.07.036 30059673

[45] Mboumba Bouassa R-S Giorgini G Silvestri C Muller C Nallabelli N Alexandrova Y. Plasma endocannabinoidome and fecal microbiota interplay in people with HIV and subclinical coronary artery disease: results from the canadian HIV and aging cohort study. iScience. (2024) 27:110456. doi: 10.1016/j.isci.2024.110456 39156649 PMC11326910

[46] Elmes MW Kaczocha M Berger WT Leung K Ralph BP Wang L. Fatty acid-binding proteins (FABPs) are intracellular carriers for Δ9-tetrahydrocannabinol (THC) and cannabidiol (CBD). J Biol Chem. (2015) 290:8711–21. doi: 10.1074/jbc.M114.618447 PMC4423662 25666611

[47] Buisseret B Alhouayek M Guillemot-Legris O Muccioli GG. Endocannabinoid and prostanoid crosstalk in pain. Trends Mol Med. (2019) 25:882–96. doi: 10.1016/j.molmed.2019.04.009 31160168

[48] Lucas CJ Galettis P Schneider J. The pharmacokinetics and the pharmacodynamics of cannabinoids. Br J Clin Pharmacol. (2018) 84:2477–82. doi: 10.1111/bcp.13710 PMC6177698 30001569

[49] Chayasirisobhon S. Mechanisms of action and pharmacokinetics of cannabis. TPJ. (2021) 25:1–3. doi: 10.7812/TPP/19.200 PMC8803256 33635755

[50] McDonnell AM Dang CH. Basic review of the cytochrome P450 system. JADPRO. (2013) 4:263–8. doi: 10.6004/jadpro.2013.4.4.7 PMC4093435 25032007

[51] Smith RT Gruber SA. Contemplating cannabis? The complex relationship between cannabinoids and hepatic metabolism resulting in the potential for drug-drug interactions. Front Psychiatry. (2023) 13:1055481. doi: 10.3389/fpsyt.2022.1055481 36704740 PMC9871609

[52] Nasrin S Watson CJW Perez-Paramo YX Lazarus P. Cannabinoid metabolites as inhibitors of major hepatic CYP450 enzymes, with implications for cannabis-drug interactions. Drug Metab Dispos. (2021) 49:1070–80. doi: 10.1124/dmd.121.000442 PMC11022895 34493602

[53] Beers JL Fu D Jackson KD. Cytochrome P450-catalyzed metabolism of cannabidiol to the active metabolite 7-hydroxy-cannabidiol. Drug Metab Dispos. (2021) 49:882–91. doi: 10.1124/dmd.120.000350 PMC11025033 34330718

[54] Cristino L Bisogno T Di Marzo V. Cannabinoids and the expanded endocannabinoid system in neurological disorders. Nat Rev Neurol. (2020) 16:9–29. doi: 10.1038/s41582-019-0284-z 31831863

[55] Mackie K. Cannabinoid receptors: where they are and what they do. J Neuroendocrinol. (2008) 20 Suppl 1:10–4. doi: 10.1111/j.1365-2826.2008.01671.x 18426493

[56] Anthony AT Rahmat S Sangle P Sandhu O Khan S. Cannabinoid receptors and their relationship with chronic pain: A narrative review. Cureus. (2020) 12:e10436. doi: 10.7759/cureus.10436 33072446 PMC7557112

[57] Zou S Kumar U. Cannabinoid receptors and the endocannabinoid system: signaling and function in the central nervous system. Int J Mol Sci. (2018) 19:833. doi: 10.3390/ijms19030833 29533978 PMC5877694

[58] Fraguas-Sánchez AI Martín-Sabroso C Torres-Suárez AI. Insights into the effects of the endocannabinoid system in cancer: A review. Br J Pharmacol. (2018) 175:2566–80. doi: 10.1111/bph.14331 PMC6003657 29663308

[59] Laprairie RB Bagher AM Kelly MEM Denovan-Wright EM. Cannabidiol is a negative allosteric modulator of the cannabinoid CB1 receptor. Br J Pharmacol. (2015) 172:4790–805. doi: 10.1111/bph.13250 PMC4621983 26218440

[60] Singh K Bhushan B Chanchal DK Sharma SK Rani K Yadav MK. Emerging therapeutic potential of cannabidiol (CBD) in neurological disorders: A comprehensive review. Behav Neurol. (2023) 2023:8825358. doi: 10.1155/2023/8825358 37868743 PMC10586905

[61] Vitale RM Iannotti FA Amodeo P. The (Poly)Pharmacology of cannabidiol in neurological and neuropsychiatric disorders: molecular mechanisms and targets. IJMS. (2021) 22:4876. doi: 10.3390/ijms22094876 34062987 PMC8124847

[62] Topol EJ Bousser M-G Fox KAA Creager MA Despres J-P Easton JD. Rimonabant for prevention of cardiovascular events (CRESCENDO): A randomised, multicentre, placebo-controlled trial. Lancet. (2010) 376:517–23. doi: 10.1016/S0140-6736(10)60935-X 20709233

[63] Jordan CJ Xi Z-X. Progress in brain cannabinoid CB2 receptor research: from genes to behavior. Neurosci Biobehav Rev. (2019) 98:208–20. doi: 10.1016/j.neubiorev.2018.12.026 PMC6401261 30611802

[64] Ma Z Gao F Larsen B Gao M Luo Z Chen D. Mechanisms of cannabinoid CB2 receptor-mediated reduction of dopamine neuronal excitability in mouse ventral tegmental area. EBioMedicine. (2019) 42:225–37. doi: 10.1016/j.ebiom.2019.03.040 PMC6491419 30952618

[65] Saroz Y Kho DT Glass M Graham ES Grimsey NL. Cannabinoid receptor 2 (CB _2_) signals via G-alpha-s and induces IL-6 and IL-10 cytokine secretion in human primary leukocytes. ACS Pharmacol Transl Sci. (2019) 2:414–28. doi: 10.1021/acsptsci.9b00049 PMC7088898 32259074

[66] Blasco-Benito S Moreno E Seijo-Vila M Tundidor I Andradas C Caffarel MM. Therapeutic targeting of HER2–CB _2_ R heteromers in HER2-positive breast cancer. Proc Natl Acad Sci USA. (2019) 116:3863–72. doi: 10.1073/pnas.1815034116 PMC6397550 30733293

[67] Scarlett KA White E-SZ Coke CJ Carter JR Bryant LK Hinton CV. Agonist-induced CXCR4 and CB2 heterodimerization inhibits Gα13/rhoA-mediated migration. Mol Cancer Res. (2018) 16:728–39. doi: 10.1158/1541-7786.MCR-16-0481 PMC5882517 29330286

[68] Ryberg E Larsson N Sjögren S Hjorth S Hermansson N Leonova J. The orphan receptor GPR55 is a novel cannabinoid receptor. Br J Pharmacol. (2007) 152:1092–101. doi: 10.1038/sj.bjp.0707460 PMC2095107 17876302

[69] Yang H Zhou J Lehmann C. GPR55 - a putative “Type 3” Cannabinoid receptor in inflammation. J Basic Clin Physiol Pharmacol. (2016) 27:297–302. doi: 10.1515/jbcpp-2015-0080 26669245

[70] Moriconi A Cerbara I Maccarrone M Topai A. GPR55: current knowledge and future perspectives of a purported “Type-3” Cannabinoid receptor. Curr Med Chem. (2010) 17:1411–29. doi: 10.2174/092986710790980069 20166924

[71] Kolbe MR Hohmann T Hohmann U Ghadban C Mackie K Zöller C. THC reduces ki67-immunoreactive cells derived from human primary glioblastoma in a GPR55-dependent manner. Cancers. (2021) 13:1064. doi: 10.3390/cancers13051064 33802282 PMC7959141

[72] Godlewski G Offertáler L Wagner JA Kunos G. Receptors for acylethanolamides-GPR55 and GPR119. Prostaglandins Other Lipid Mediat. (2009) 89:105–11. doi: 10.1016/j.prostaglandins.2009.07.001 PMC2751869 19615459

[73] He Y Shen H Bi G-H Zhang H-Y Soler-Cedeño O Alton H. GPR55 is expressed in glutamate neurons and functionally modulates drug taking and seeking in rats and mice. Transl Psychiatry. (2024) 14:101. doi: 10.1038/s41398-024-02820-3 38374108 PMC10876975

[74] Rosenberg EC Chamberland S Bazelot M Nebet ER Wang X McKenzie S. Cannabidiol modulates excitatory-inhibitory ratio to counter hippocampal hyperactivity. Neuron. (2023) 111:1282–1300.e8. doi: 10.1016/j.neuron.2023.01.018 36787750 PMC12243023

[75] Gasperi V Dainese E Oddi S Sabatucci A Maccarrone M. GPR55 and its interaction with membrane lipids: comparison with other endocannabinoid-binding receptors. Curr Med Chem. (2013) 20:64–78. doi: 10.2174/0929867311302010008 23151004

[76] Saliba SW Jauch H Gargouri B Keil A Hurrle T Volz N. Anti-neuroinflammatory effects of GPR55 antagonists in LPS-activated primary microglial cells. J Neuroinflamm. (2018) 15:322. doi: 10.1186/s12974-018-1362-7 PMC6240959 30453998

[77] Mishiro S Igarashi-Takeuchi H Numabe Y. Expression of cannabinoid receptor GPR55 in human gingival fibroblasts and anti-inflammatory effects of cannabidiol via GPR55. Nihon Shishubyo Gakkai Kaishi. (2021) 63:11–23. doi: 10.2329/perio.63.11

[78] Lah TT Majc B Novak M Sušnik A Breznik B Porčnik A. The cytotoxic effects of cannabidiol and cannabigerol on glioblastoma stem cells may mostly involve GPR55 and TRPV1 signalling. Cancers (Basel). (2022) 14:5918. doi: 10.3390/cancers14235918 36497400 PMC9738061

[79] Ito A Omi J Aoki J. Inositolphospholipids and GPR55. BPB Rep. (2024) 7:90–5. doi: 10.1248/bpbreports.7.3_90

[80] Honkisz-Orzechowska E Łażewska D Baran G Kieć-Kononowicz K. Uncovering the power of GPR18 signalling: how rvD2 and other ligands could have the potential to modulate and resolve inflammation in various health disorders. Molecules. (2024) 29:1258. doi: 10.3390/molecules29061258 38542895 PMC10976181

[81] Liu Y Wang L Lo K-W Lui VWY. Omics-wide quantitative B-cell infiltration analyses identify GPR18 for human cancer prognosis with superiority over CD20. Commun Biol. (2020) 3:234. doi: 10.1038/s42003-020-0964-7 32398659 PMC7217858

[82] Zhang M Ma Y Ye X Zhang N Pan L Wang B. TRP (Transient receptor potential) ion channel family: structures, biological functions and therapeutic interventions for diseases. Sig Transduct Target Ther. (2023) 8:261. doi: 10.1038/s41392-023-01464-x PMC10319900 37402746

[83] Muller C Morales P Reggio PH. Cannabinoid ligands targeting TRP channels. Front Mol Neurosci. (2019) 11:487. doi: 10.3389/fnmol.2018.00487 30697147 PMC6340993

[84] Marini M Titiz M Souza Monteiro de Araújo D Geppetti P Nassini R De Logu F. TRP channels in cancer: signaling mechanisms and translational approaches. Biomolecules. (2023) 13:1557. doi: 10.3390/biom13101557 37892239 PMC10605459

[85] Bai S Wei Y Liu R Chen Y Ma W Wang M. The role of transient receptor potential channels in metastasis. Biomedicine Pharmacotherapy. (2023) 158:114074. doi: 10.1016/j.biopha.2022.114074 36493698

[86] De La Harpe A Beukes N Frost CL. CBD activation of TRPV1 induces oxidative signaling and subsequent ER stress in breast cancer cell lines. Biotech App Biochem. (2022) 69:420–30. doi: 10.1002/bab.2119 33604949

[87] Liu S Wang Q Li Z Ma L Li T Li Y. TRPV1 channel activated by the PGE2/EP4 pathway mediates spinal hypersensitivity in a mouse model of vertebral endplate degeneration. Oxid Med Cell Longevity. (2021) 2021:1–16. doi: 10.1155/2021/9965737 PMC8405310 34471470

[88] Qin N Neeper MP Liu Y Hutchinson TL Lubin ML Flores CM. TRPV2 is activated by cannabidiol and mediates CGRP release in cultured rat dorsal root ganglion neurons. J Neurosci. (2008) 28:6231–8. doi: 10.1523/JNEUROSCI.0504-08.2008 PMC6670541 18550765

[89] Su W Qiao X Wang W He S Liang K Hong X. TRPV3: structure, diseases and modulators. Molecules. (2023) 28:774. doi: 10.3390/molecules28020774 36677834 PMC9865980

[90] Michalick L Kuebler WM. TRPV4—A missing link between mechanosensation and immunity. Front Immunol. (2020) 11:413. doi: 10.3389/fimmu.2020.00413 32210976 PMC7076180

[91] Antoniazzi CTDD Ruviaro NA Peres DS Rodrigues P Viero FT Trevisan G. Targeting TRPV4 channels for cancer pain relief. Cancers. (2024) 16:1703. doi: 10.3390/cancers16091703 38730655 PMC11083562

[92] Ochoa SV Casas Z Albarracín SL Sutachan JJ Torres YP. Therapeutic potential of TRPM8 channels in cancer treatment. Front Pharmacol. (2023) 14:1098448. doi: 10.3389/fphar.2023.1098448 37033630 PMC10073478

[93] Asuthkar S Demirkhanyan L Sun X Elustondo PA Krishnan V Baskaran P. The TRPM8 protein is a testosterone receptor. J Biol Chem. (2015) 290:2670–88. doi: 10.1074/jbc.M114.610873 PMC4316998 25480785

[94] Takahashi N Chen H-Y Harris IS Stover DG Selfors LM Bronson RT. Cancer cells co-opt the neuronal redox-sensing channel TRPA1 to promote oxidative-stress tolerance. Cancer Cell. (2018) 33:985–1003.e7. doi: 10.1016/j.ccell.2018.05.001 29805077 PMC6100788

[95] Shoshan-Barmatz V Shteinfer-Kuzmine A Verma A. VDAC1 at the intersection of cell metabolism, apoptosis, and diseases. Biomolecules. (2020) 10:1485. doi: 10.3390/biom10111485 33114780 PMC7693975

[96] Mahmoud AM Kostrzewa M Marolda V Cerasuolo M Maccarinelli F Coltrini D. Cannabidiol alters mitochondrial bioenergetics via VDAC1 and triggers cell death in hormone-refractory prostate cancer. Pharmacol Res. (2023) 189:106683. doi: 10.1016/j.phrs.2023.106683 36736415

[97] Malheiro RF Carmo H Carvalho F Silva JP. Cannabinoid-mediated targeting of mitochondria on the modulation of mitochondrial function and dynamics. Pharmacol Res. (2023) 187:106603. doi: 10.1016/j.phrs.2022.106603 36516885

[98] O’Sullivan SE. An update on PPAR activation by cannabinoids. Br J Pharmacol. (2016) 173:1899–910. doi: 10.1111/bph.13497 PMC4882496 27077495

[99] Vara D Morell C Rodríguez-Henche N Diaz-Laviada I. Involvement of PPARγ in the antitumoral action of cannabinoids on hepatocellular carcinoma. Cell Death Dis. (2013) 4:e618. doi: 10.1038/cddis.2013.141 23640460 PMC3674350

[100] Hartley A Ahmad I. The role of PPARγ in prostate cancer development and progression. Br J Cancer. (2023) 128:940–5. doi: 10.1038/s41416-022-02096-8 PMC10006070 36510001

[101] Alsalem M Haddad M Aldossary SA Kalbouneh H Altarifi A Jaffal SM. Role of cannabinoid receptor 1 and the peroxisome proliferator-activated receptor α in mediating anti-nociceptive effects of synthetic cannabinoids and a cannabinoid-like compound. Inflammopharmacol. (2019) 27:1131–42. doi: 10.1007/s10787-019-00584-7 30945071

[102] Ghasemi-Gojani E Kovalchuk I Kovalchuk O. Cannabinoids and terpenes for diabetes mellitus and its complications: from mechanisms to new therapies. Trends Endocrinol Metab. (2022) 33:828–49. doi: 10.1016/j.tem.2022.08.003 36280497

[103] Sarrouilhe D Mesnil M. Serotonin and human cancer: A critical view. Biochimie. (2019) 161:46–50. doi: 10.1016/j.biochi.2018.06.016 29936294

[104] Wright NJD. A review of the direct targets of the cannabinoids cannabidiol, Δ9-tetrahydrocannabinol, N-arachidonoylethanolamine and 2-arachidonoylglycerol. AIMSN. (2024) 11:144–65. doi: 10.3934/Neuroscience.2024009 PMC11230856 38988890

[105] Martínez-Aguirre C Carmona-Cruz F Velasco AL Velasco F Aguado-Carrillo G Cuéllar-Herrera M. Cannabidiol acts at 5-HT1A receptors in the human brain: relevance for treating temporal lobe epilepsy. Front Behav Neurosci. (2020) 14:611278. doi: 10.3389/fnbeh.2020.611278 33384591 PMC7770178

[106] Billard E Torbey A Inserra A Grant E Bertazzo A De Gregorio D. Pharmacological characterization of cannabidiol as a negative allosteric modulator of the 5-HT2A receptor. Cell Signalling. (2025) 127:111588. doi: 10.1016/j.cellsig.2025.111588 39761844

[107] Xiong W Cui T Cheng K Yang F Chen S-R Willenbring D. Cannabinoids suppress inflammatory and neuropathic pain by targeting α3 glycine receptors. J Exp Med. (2012) 209:1121–34. doi: 10.1084/jem.20120242 PMC3371734 22585736

[108] Förstera B Dzaye O Winkelmann A Semtner M Benedetti B Markovic DS. Intracellular glycine receptor function facilitates glioma formation *in vivo* . J Cell Sci. (2014) 127:3687–98. doi: 10.1242/jcs.146662 24994934

[109] Blanke ML VanDongen AMJ. Activation Mechanisms of the NMDA Receptor. In: Van Dongen AM, editor. Biology of the NMDA Receptor. CRC Press/Taylor & Francis, Boca Raton (FL (2009). 21204408

[110] Sánchez-Blázquez P Rodríguez-Muñoz M Garzón J. The cannabinoid receptor 1 associates with NMDA receptors to produce glutamatergic hypofunction: implications in psychosis and schizophrenia. Front Pharmacol. (2014) 4:169. doi: 10.3389/fphar.2013.00169 24427139 PMC3877778

[111] Rivas-Santisteban R Lillo A Lillo J Rebassa J-B Contestí JS Saura CA. N-methyl-D-aspartate (NMDA) and cannabinoid CB2 receptors form functional complexes in cells of the central nervous system: insights into the therapeutic potential of neuronal and microglial NMDA receptors. Alzheimers Res Ther. (2021) 13:184. doi: 10.1186/s13195-021-00920-6 34749800 PMC8576920

[112] Esteban-Martínez L Torres M. Metabolic regulation of cell competition. Dev Biol. (2021) 475:30–6. doi: 10.1016/j.ydbio.2021.02.011 33652024

[113] Gallo S Vitacolonna A Crepaldi T. NMDA receptor and its emerging role in cancer. Int J Mol Sci. (2023) 24:2540. doi: 10.3390/ijms24032540 36768862 PMC9917092

[114] Castillo A Tolón MR Fernández-Ruiz J Romero J Martinez-Orgado J. The neuroprotective effect of cannabidiol in an *in vitro* model of newborn hypoxic–ischemic brain damage in mice is mediated by CB2 and adenosine receptors. Neurobiol Dis. (2010) 37:434–40. doi: 10.1016/j.nbd.2009.10.023 19900555

[115] De Fátima Dos Santos Sampaio M De Paiva YB Sampaio TB Pereira MG Coimbra NC. Therapeutic applicability of cannabidiol and other phytocannabinoids in epilepsy, multiple sclerosis and parkinson’s disease and in comorbidity with psychiatric disorders. Basic Clin Pharma Tox. (2024) 134:574–601. doi: 10.1111/bcpt.13997 38477419

[116] Carriba P Ortiz O Patkar K Justinova Z Stroik J Themann A. Striatal adenosine A2A and cannabinoid CB1 receptors form functional heteromeric complexes that mediate the motor effects of cannabinoids. Neuropsychopharmacol. (2007) 32:2249–59. doi: 10.1038/sj.npp.1301375 17356572

[117] Moreno E Cavic M Krivokuca A Casadó V Canela E. The endocannabinoid system as a target in cancer diseases: are we there yet? Front Pharmacol. (2019) 10:339. doi: 10.3389/fphar.2019.00339 31024307 PMC6459931

[118] Jarrahian A Watts VJ Barker EL. D2 dopamine receptors modulate galpha-subunit coupling of the CB1 cannabinoid receptor. J Pharmacol Exp Ther. (2004) 308:880–6. doi: 10.1124/jpet.103.057620 14634050

[119] Sun D Li X Nie S Liu J Wang S. Disorders of cancer metabolism: the therapeutic potential of cannabinoids. Biomedicine Pharmacotherapy. (2023) 157:113993. doi: 10.1016/j.biopha.2022.113993 36379120

[120] Hinz B Ramer R. Cannabinoids as anticancer drugs: current status of preclinical research. Br J Cancer. (2022) 127:1–13. doi: 10.1038/s41416-022-01727-4 35277658 PMC9276677

[121] Dariš B Tancer Verboten M Knez Ž Ferk P. Cannabinoids in cancer treatment: therapeutic potential and legislation. Bosn J Basic Med Sci. (2019) 19:14–23. doi: 10.17305/bjbms.2018.3532 30172249 PMC6387667

[122] Xiong X Chen S Shen J You H Yang H Yan C. Cannabis suppresses antitumor immunity by inhibiting JAK/STAT signaling in T cells through CNR2. Signal Transduct Target Ther. (2022) 7:99. doi: 10.1038/s41392-022-00918-y 35383142 PMC8983672

[123] Sarsembayeva A Kienzl M Gruden E Ristic D Maitz K Valadez-Cosmes P. Cannabinoid receptor 2 plays a pro-tumorigenic role in non-small cell lung cancer by limiting anti-tumor activity of CD8+ T and NK cells. Front Immunol. (2022) 13:997115. doi: 10.3389/fimmu.2022.997115 36700219 PMC9868666

[124] Waissengrin B Leshem Y Taya M Meiri D Merimsky O Shamai S. The use of medical cannabis concomitantly with immune checkpoint inhibitors in non-small cell lung cancer: A sigh of relief? Eur J Cancer. (2023) 180:52–61. doi: 10.1016/j.ejca.2022.11.022 36535195

[125] Taha T Meiri D Talhamy S Wollner M Peer A Bar-Sela G. Cannabis impacts tumor response rate to nivolumab in patients with advanced Malignancies. Oncologist. (2019) 24:549–54. doi: 10.1634/theoncologist.2018-0383 PMC6459234 30670598

[126] Bar-Sela G Cohen I Campisi-Pinto S Lewitus GM Oz-Ari L Jehassi A. Cannabis consumption used by cancer patients during immunotherapy correlates with poor clinical outcome. Cancers (Basel). (2020) 12:2447. doi: 10.3390/cancers12092447 32872248 PMC7563978

[127] Biedny A Szpunar S Abdalla A Kafri Z Hadid TH. The effect of concomitant cannabinoids during immune checkpoint inhibitor treatment of advanced stage Malignancy. JCO. (2020) 38:e15064–4. doi: 10.1200/JCO.2020.38.15_suppl.e15064

[128] Sun X Zhou L Wang Y Deng G Cao X Ke B. Single-cell analyses reveal cannabidiol rewires tumor microenvironment via inhibiting alternative activation of macrophage and synergizes with anti-PD-1 in colon cancer. J Pharm Anal. (2023) 13:726–44. doi: 10.1016/j.jpha.2023.04.013 PMC10422166 37577382

[129] Kim BG Kim BR Kim DY Kim WY Kang S Lee SI. Cannabidiol Enhances Atezolizumab Efficacy by Upregulating PD-L1 Expression via the cGAS–STING Pathway in Triple-Negative Breast Cancer Cells. Cancer Immunol Res. (2024) 12:1796–807. doi: 10.1158/2326-6066.CIR-23-0902 PMC11612622 39226389

[130] Yang Y Huynh N Dumesny C Wang K He H Nikfarjam M. Cannabinoids inhibited pancreatic cancer via P-21 activated kinase 1 mediated pathway. IJMS. (2020) 21:8035. doi: 10.3390/ijms21218035 33126623 PMC7662796

[131] Centanni M Moes DJAR Trocóniz IF Ciccolini J van Hasselt JGC. Clinical pharmacokinetics and pharmacodynamics of immune checkpoint inhibitors. Clin Pharmacokinet. (2019) 58:835–57. doi: 10.1007/s40262-019-00748-2 PMC6584248 30815848

[132] Cherkasova V Wang B Gerasymchuk M Fiselier A Kovalchuk O Kovalchuk I. Use of cannabis and cannabinoids for treatment of cancer. Cancers. (2022) 14:5142. doi: 10.3390/cancers14205142 36291926 PMC9600568

[133] Gustafsson SB Palmqvist R Henriksson ML Dahlin AM Edin S Jacobsson SOP. High tumour cannabinoid CB1 receptor immunoreactivity negatively impacts disease-specific survival in stage II microsatellite stable colorectal cancer. PloS One. (2011) 6:e23003. doi: 10.1371/journal.pone.0023003 21901119 PMC3161987

[134] Foyzun T Whiting M Velasco KK Jacobsen JC Connor M Grimsey NL. Single nucleotide polymorphisms in the cannabinoid CB _2_ receptor: molecular pharmacology and disease associations. Br J Pharmacol. (2024) 181:2391–412. doi: 10.1111/bph.16383 38802979

[135] Doris JM Millar SA Idris I O’Sullivan SE. Genetic polymorphisms of the endocannabinoid system in obesity and diabetes. Diabetes Obes Metab. (2019) 21:382–7. doi: 10.1111/dom.13504 30129173

[136] Li T Chung M-K. Striving toward hyperthermia-free analgesia: lessons from loss-of-function mutations of human TRPV1. J Clin Invest. (2023) 133:e167338. doi: 10.1172/JCI167338 36719371 PMC9888373

[137] Lazary J Eszlari N Kriko E Tozser D Dome P Deakin JFW. Genetic analyses of the endocannabinoid pathway in association with affective phenotypic variants. Neurosci Lett. (2021) 744:135600. doi: 10.1016/j.neulet.2020.135600 33421489

[138] Ağagündüz D Cocozza E Cemali Ö Bayazıt AD Nanì MF Cerqua I. Understanding the role of the gut microbiome in gastrointestinal cancer: A review. Front Pharmacol. (2023) 14:1130562. doi: 10.3389/fphar.2023.1130562 36762108 PMC9903080

[139] Xia C Su J Liu C Mai Z Yin S Yang C. Human microbiomes in cancer development and therapy. MedComm. (2023) 4:e221. doi: 10.1002/mco2.221 36860568 PMC9969057

[140] Ahn J Sinha R Pei Z Dominianni C Wu J Shi J. Human gut microbiome and risk for colorectal cancer. J Natl Cancer Inst. (2013) 105:1907–11. doi: 10.1093/jnci/djt300 PMC3866154 24316595

[141] Rescigno M. Training the microbiota to increase immune checkpoint blockade and to reduce toxicity. Eur J Immunol. (2023) 53:e2250183. doi: 10.1002/eji.202250183 36747375

[142] Davar D Dzutsev AK McCulloch JA Rodrigues RR Chauvin J-M Morrison RM. Fecal microbiota transplant overcomes resistance to anti–PD-1 therapy in melanoma patients. Science. (2021) 371:595–602. doi: 10.1126/science.abf3363 33542131 PMC8097968

[143] Cohen LJ Esterhazy D Kim S-H Lemetre C Aguilar RR Gordon EA. Commensal bacteria make GPCR ligands that mimic human signalling molecules. Nature. (2017) 549:48–53. doi: 10.1038/nature23874 28854168 PMC5777231

[144] Depommier C Vitale RM Iannotti FA Silvestri C Flamand N Druart C. Beneficial effects of akkermansia muciniphila are not associated with major changes in the circulating endocannabinoidome but linked to higher mono-palmitoyl-glycerol levels as new PPARα Agonists. Cells. (2021) 10:185. doi: 10.3390/cells10010185 33477821 PMC7832901

[145] Al-Khazaleh AK Jaye K Chang D Münch GW Bhuyan DJ. Buds and bugs: A fascinating tale of gut microbiota and cannabis in the fight against cancer. Int J Mol Sci. (2024) 25:872. doi: 10.3390/ijms25020872 38255944 PMC10815411

[146] Srivastava RK Lutz B Ruiz De Azua I. The microbiome and gut endocannabinoid system in the regulation of stress responses and metabolism. Front Cell Neurosci. (2022) 16:867267. doi: 10.3389/fncel.2022.867267 35634468 PMC9130962

[147] McDew-White M Lee E Premadasa LS Alvarez X Okeoma CM Mohan M. Cannabinoids modulate the microbiota–gut–brain axis in HIV/SIV infection by reducing neuroinflammation and dysbiosis while concurrently elevating endocannabinoid and indole-3-propionate levels. J Neuroinflamm. (2023) 20:62. doi: 10.1186/s12974-023-02729-6 PMC9993397 36890518

[148] Vitetta L Nation T Oldfield D Thomsen M. Medicinal cannabis and the intestinal microbiome. Pharm (Basel). (2024) 17:1702. doi: 10.3390/ph17121702 PMC11678570 39770543

[149] Stella B Baratta F Della Pepa C Arpicco S Gastaldi D Dosio F. Cannabinoid formulations and delivery systems: current and future options to treat pain. Drugs. (2021) 81:1513–57. doi: 10.1007/s40265-021-01579-x PMC8417625 34480749

[150] Reddy TS Zomer R Mantri N. Nanoformulations as a strategy to overcome the delivery limitations of cannabinoids. Phytotherapy Res. (2023) 37:1526–38. doi: 10.1002/ptr.7742 36748949

[151] Konstandi M Johnson EO. Age-related modifications in CYP-dependent drug metabolism: role of stress. Front Endocrinol. (2023) 14:1143835. doi: 10.3389/fendo.2023.1143835 PMC10244505 37293497

[152] Parihar V Rogers A Blain AM Zacharias SRK Patterson LL Siyam MA-M. Reduction in tamoxifen metabolites endoxifen and N-desmethyltamoxifen with chronic administration of low dose cannabidiol: A CYP3A4 and CYP2D6 drug interaction. J Pharm Pract. (2022) 35:322–6. doi: 10.1177/0897190020972208 33191836

[153] Schoeman R de la Harpe A Beukes N Frost CL. Cannabis with breast cancer treatment: propitious or pernicious? 3 Biotech. (2022) 12:54. doi: 10.1007/s13205-021-03102-1 PMC8807790 35127309

[154] Olivas-Aguirre M Torres-López L Gómez-Sandoval Z Villatoro-Gómez K Pottosin I Dobrovinskaya O. Tamoxifen sensitizes acute lymphoblastic leukemia cells to cannabidiol by targeting cyclophilin-D and altering mitochondrial ca2+ Homeostasis. IJMS. (2021) 22:8688. doi: 10.3390/ijms22168688 34445394 PMC8395529

[155] Go YY Kim SR Kim DY Chae S-W Song J-J. Cannabidiol enhances cytotoxicity of anti-cancer drugs in human head and neck squamous cell carcinoma. Sci Rep. (2020) 10:20622. doi: 10.1038/s41598-020-77674-y 33244087 PMC7692486

[156] Qian Y Gurley BJ Markowitz JS. The potential for pharmacokinetic interactions between cannabis products and conventional medications. J Clin Psychopharmacol. (2019) 39:462–71. doi: 10.1097/JCP.0000000000001089 31433338

[157] Takheaw N Jindaphun K Pata S Laopajon W Kasinrerk W. Cannabinoid receptor 1 agonist ACEA and cannabinoid receptor 2 agonist GW833972A attenuates cell-mediated immunity by different biological mechanisms. Cells. (2023) 12:848. doi: 10.3390/cells12060848 36980189 PMC10047765

[158] Robinson RH Meissler JJ Fan X Yu D Adler MW Eisenstein TKA. CB2-selective cannabinoid suppresses T-cell activities and increases tregs and IL-10. J Neuroimmune Pharmacol. (2015) 10:318–32. doi: 10.1007/s11481-015-9611-3 PMC4528965 25980325

[159] Henriquez JE Bach AP Matos-Fernandez KM Crawford RB Kaminski NE. [amp]]Delta;9-tetrahydrocannabinol (THC) impairs CD8+ T cell-mediated activation of astrocytes. J Neuroimmune Pharmacol. (2020) 15:863–74. doi: 10.1007/s11481-020-09912-z PMC7529688 32215844

[160] Eisenstein TK Meissler JJ. Effects of cannabinoids on T-cell function and resistance to infection. J Neuroimmune Pharmacol. (2015) 10:204–16. doi: 10.1007/s11481-015-9603-3 PMC4470840 25876735

[161] Ivanova M Shivarov V. HLA genotyping meets response to immune checkpoint inhibitors prediction: A story just started. Int J Immunogenetics. (2021) 48:193–200. doi: 10.1111/iji.12517 33112034

[162] Dada S Ellis SLS Wood C Nohara LL Dreier C Garcia NH. Specific cannabinoids revive adaptive immunity by reversing immune evasion mechanisms in metastatic tumours. Front Immunol. (2023) 13:982082. doi: 10.3389/fimmu.2022.982082 36923728 PMC10010394

[163] Sarsembayeva A Schicho R. Cannabinoids and the endocannabinoid system in immunotherapy: helpful or harmful? Front Oncol. (2023) 13:1296906. doi: 10.3389/fonc.2023.1296906 38074691 PMC10699860

[164] Kienzl M Hasenoehrl C Maitz K Sarsembayeva A Taschler U Valadez-Cosmes P. Monoacylglycerol lipase deficiency in the tumor microenvironment slows tumor growth in non-small cell lung cancer. OncoImmunology. (2021) 10:1965319. doi: 10.1080/2162402X.2021.1965319 34527428 PMC8437460

[165] Lei Q Wang D Sun K Wang L Zhang Y. Resistance mechanisms of anti-PD1/PDL1 therapy in solid tumors. Front Cell Dev Biol. (2020) 8:672. doi: 10.3389/fcell.2020.00672 32793604 PMC7385189

[166] Miralpeix C Reguera AC Fosch A Zagmutt S Casals N Cota D. Hypothalamic endocannabinoids in obesity: an old story with new challenges. Cell Mol Life Sci. (2021) 78:7469–90. doi: 10.1007/s00018-021-04002-6 PMC8557709 34718828

[167] Scotchie JG Savaris RF Martin CE Young SL. Endocannabinoid regulation in human endometrium across the menstrual cycle. Reprod Sci. (2015) 22:113–23. doi: 10.1177/1933719114533730 PMC4527420 24819878

[168] Toczek M Malinowska B. Enhanced endocannabinoid tone as a potential target of pharmacotherapy. Life Sci. (2018) 204:20–45. doi: 10.1016/j.lfs.2018.04.054 29729263

[169] Lu Y Anderson HD. Cannabinoid signaling in health and disease. Can J Physiol Pharmacol. (2017) 95:311–27. doi: 10.1139/cjpp-2016-0346 28263083

[170] Patel TH Brewer JR Fan J Cheng J Shen Y-L Xiang Y. FDA approval summary: tremelimumab in combination with durvalumab for the treatment of patients with unresectable hepatocellular carcinoma. Clin Cancer Res. (2024) 30:269–73. doi: 10.1158/1078-0432.CCR-23-2124 PMC10841291 37676259

[171] Khair DO Bax HJ Mele S Crescioli S Pellizzari G Khiabany A. Combining immune checkpoint inhibitors: established and emerging targets and strategies to improve outcomes in melanoma. Front Immunol. (2019) 10:453. doi: 10.3389/fimmu.2019.00453 30941125 PMC6435047

[172] Akinleye A Rasool Z. Immune checkpoint inhibitors of PD-L1 as cancer therapeutics. J Hematol Oncol. (2019) 12:92. doi: 10.1186/s13045-019-0779-5 31488176 PMC6729004

[173] Friedlaender A Addeo A Banna G. New emerging targets in cancer immunotherapy: the role of TIM3. ESMO Open. (2019) 4:e000497. doi: 10.1136/esmoopen-2019-000497 31275616 PMC6579568

[174] Chauvin J-M Zarour HM. TIGIT in cancer immunotherapy. J Immunother Cancer. (2020) 8:e000957. doi: 10.1136/jitc-2020-000957 32900861 PMC7477968

[175] Maruhashi T Sugiura D Okazaki I Okazaki T. LAG-3: from molecular functions to clinical applications. J Immunother Cancer. (2020) 8:e001014. doi: 10.1136/jitc-2020-001014 32929051 PMC7488795

[176] Martin AS Molloy M Ugolkov A Von Roemeling RW Noelle RJ Lewis LD. VISTA expression and patient selection for immune-based anticancer therapy. Front Immunol. (2023) 14:1086102. doi: 10.3389/fimmu.2023.1086102 36891296 PMC9986543

[177] Ta HM Roy D Zhang K Alban T Juric I Dong J. LRIG1 engages ligand VISTA and impairs tumor-specific CD8+ T cell responses. Sci Immunol. (2024) 9:eadi7418. doi: 10.1126/sciimmunol.adi7418 38758807 PMC11334715

[178] Olbromski M Mrozowska M Piotrowska A Smolarz B Romanowicz H. The VISTA/VSIG3/PSGL-1 axis: crosstalk between immune effector cells and cancer cells in invasive ductal breast carcinoma. Cancer Immunol Immunother. (2024) 73:136. doi: 10.1007/s00262-024-03701-w 38833004 PMC11150347

[179] Poggi A Zocchi MR. Natural killer cells and immune-checkpoint inhibitor therapy: current knowledge and new challenges. Mol Ther - Oncolytics. (2022) 24:26–42. doi: 10.1016/j.omto.2021.11.016 34977340 PMC8693432

[180] Wang X Xiong H Ning Z. Implications of NKG2A in immunity and immune-mediated diseases. Front Immunol. (2022) 13:960852. doi: 10.3389/fimmu.2022.960852 36032104 PMC9399941

[181] Tian J Ashique AM Weeks S Lan T Yang H Chen H-IH. ILT2 and ILT4 drive myeloid suppression via both overlapping and distinct mechanisms. Cancer Immunol Res. (2024) 12:592–613. doi: 10.1158/2326-6066.CIR-23-0568 38393969

[182] Mandel I Haves Ziv D Goldshtein I Peretz T Alishekevitz D Fridman Dror A. BND-22, a first-in-class humanized ILT2-blocking antibody, promotes antitumor immunity and tumor regression. J Immunother Cancer. (2022) 10:e004859. doi: 10.1136/jitc-2022-004859 36096532 PMC9472153

[183] Hu Z Zhang Q He Z Jia X Zhang W Cao X. MHC1/LILRB1 axis as an innate immune checkpoint for cancer therapy. Front Immunol. (2024) 15:1421092. doi: 10.3389/fimmu.2024.1421092 38911856 PMC11190085

[184] Li X Tian W Jiang Z Song Y Leng X Yu J. Targeting CD24/siglec-10 signal pathway for cancer immunotherapy: recent advances and future directions. Cancer Immunol Immunother. (2024) 73:31. doi: 10.1007/s00262-023-03606-0 38279998 PMC10821995

[185] Zhao K Wu C Li X Niu M Wu D Cui X. From mechanism to therapy: the journey of CD24 in cancer. Front Immunol. (2024) 15:1401528. doi: 10.3389/fimmu.2024.1401528 38881902 PMC11176514

[186] Berrazouane S Boisvert M Salti S Mourad W Al-Daccak R Barabé F. Beta1 integrin blockade overcomes doxorubicin resistance in human T-cell acute lymphoblastic leukemia. Cell Death Dis. (2019) 10:357. doi: 10.1038/s41419-019-1593-2 31043590 PMC6494825

[187] Rosenbaum T Morales-Lázaro SL Islas LD. TRP channels: A journey towards a molecular understanding of pain. Nat Rev Neurosci. (2022) 23:596–610. doi: 10.1038/s41583-022-00611-7 35831443

